# Exploring the Impact of Child Hospitalisation on the Family System: A Qualitative Study Using Framework Analysis

**DOI:** 10.3390/children12091159

**Published:** 2025-08-31

**Authors:** Lauren Murray, Nicola Doherty, Pauline Adair

**Affiliations:** 1 School of Psychology, Queen’s University Belfast, David Keir Building, Malone Road, Belfast BT9 5BP, Northern Ireland, UK; p.adair@qub.ac.uk; 2 Regional Trauma Network, Department of Health, Gransha Park, Derry BT47 6FN, Northern Ireland, UK; nicola.doherty@hscni.net; 3 School of Psychology, Ulster University, Cromore Road, Coleraine BT52 1SA, Northern Ireland, UK

**Keywords:** family system, implications, child hospitalisation, experiences, framework analysis

## Abstract

Background: Child hospitalisation can have emotional, practical, financial, and social sequalae for the family. By understanding the impact and challenges involved, this research can inform clinical practice and interventions to help mitigate the impacts of hospitalisation. Exploring the experiences and realities of child hospitalisation for main caregivers, extended caregivers, and child siblings is the focus of this research. Method: Semi-structured interviews were designed and conducted. Eight families participated in the research. Three interviews were conducted per family, with a total of twenty-three interviews conducted overall. Interviews were recorded using Microsoft Teams application. Analysis: The interviews underwent analysis employing the Framework Method, uncovering patterns and insights relevant to the impact of child hospitalisation from varying familial perspectives. Results: Overarching themes of emotional impact, relational impact, practicalities, adjustment, communication, and “take-home messages from families” were identified and discussed. Conclusions: By identifying gaps in support, communication barriers, access inequalities, and other implications, targeted clinical interventions and preventions can be developed to empower families and healthcare professionals. This study promotes a greater understanding of the challenges associated with child hospitalisation and signifies the importance of restructuring support systems worldwide.

## 1. Introduction

Existing research on child hospitalisation primarily focuses on the perspectives of children with Chronic Health Conditions (CHCs) and their primary caregivers during periods of hospitalisation [1]. Early research in this area illuminated gaps in knowledge within the experiences of CHC-affected families, reinforcing the importance of broadening the scope of exploration [1,2,3]. Subsequent research sought to bridge these gaps by delving into the perspectives of siblings of hospitalised children [4,5,6]. However, a stark limitation of the existing literature lies in its narrow focus of CHCs, predominately focusing on specific medical conditions or diagnoses [7]. This limited scope hinders the applicability of the findings across CHCs, highlighting the need for more varied research. These gaps and limitations influenced the innovative directions of this study by exploring the familial impacts of varied CHCs from the perspectives of main caregivers, extended family caregivers, and child siblings. By addressing this gap, the research aims to provide a comprehensive understanding of the impacts of hospitalisation on the entire family unit, regardless of the nature or severity of the CHC, to support the development of more inclusive interventions and support services at local, regional, national, and global levels, thereby contributing to evolvements in paediatric healthcare worldwide.

Copious research posits child hospitalisation to be an extremely challenging and traumatic experience for the family [8,9,10,11,12]. Family members, especially caregivers, often feel overwhelmed with emotional, physical and financial stressors of the hospital experience [10,11,12,13,14]. Findings from a recent survey [15] revealed that 75% of participants experienced a negative impact on their mental health, while 50% reported a negative impact on their physical health. Caregivers do attempt to seek mental health supports to cope with the impacts of hospitalisation according to the Children in Hospital (CiHI) survey [13]. This found that 21% of caregivers attempted to access mental health services to address their psychological distress. Among those who did not seek support, 23.1% cited lack of affordability as a barrier to accessing services [13]. This is an important finding, as existing research [12] revealed that caregivers of children necessitating frequent hospitalisations often endure heightened levels of stress, anxiety, and depression, persisting for up to three months post-discharge from hospital [10,12]. This suggests ingrained and enduring emotional implications from the hospitalisation experience for caregivers.

Influential findings from various studies indicate that stressors, such as the necessity to sleep in unfamiliar hospital accommodation and the emotional strain of being separated from partners or other children, significantly impacts caregivers [13,16,17]. These findings illuminate the complex nature of caring for children with CHCs, highlighting the need for tailored supports to address caregivers’ diverse needs. Familial separation can have psychosocial and behavioural implications, including increased anxiety and loneliness [18,19]. Sibling children separated from their caregivers in this way receive much of their care from others, such as fathers, grandparents, or other friends and relatives; thus, gaining their perspectives of family hospitalisation is imperative. In attempts to address this gap in the literature, Niinomi and Fukui (2022) explored the perspectives of mothers and those of individuals who assumed caregiving duties for siblings during periods of hospitalisation. Caregivers perceived the child sibling’s distress in many ways, such as seeking comfort in familiar people and environments, seeking a sense of normalcy in their daily lives, and managing their distress by internalising and externalising difficulties [18]. Additionally, the research highlighted the potential risks associated with siblings internalising their challenges, manifesting in withdrawal, somatic complaints, anxiety, and depression, consistent with prior findings [18,20]. However, the study revealed discrepancies between observations made by mothers and those made by other caregivers, indicating variations in perceptions and experiences within the family unit. This highlights the importance of recognising diverse perspectives in the family system, supporting the need for this research.

Acknowledging child siblings as important members of the family, each with their own unique needs, is essential for the implementation of effective support interventions and represents a critical area for further research. The hospitalisation experience may inadvertently lead to child siblings’ needs being overlooked, thereby adversely affecting their physical, emotional, and relational well-being [21]. Siblings may struggle to comprehend the sudden changes in their family dynamics and separation, cope with feelings of neglect or jealousy, and grapple with the fear of losing their sibling. The disruption to daily routines, decreased parental availability, and changes in family dynamics can further exacerbate siblings’ distress and affect their psychosocial well-being [21,22]. This sheds light onto the ripple effects of caregiving challenges on various facets of family life. Similarly, Hilário (2022) emphasised the familial roles that siblings of a CHC child can adopt, specifically displaying carer responsibilities [23]. Their review examined sibling carer roles and responsibilities, revealing that a shift in caregiving roles may result in burdensome experiences for the sibling and detrimentally affect their overall well-being [23]. The review further identified that such changes could be regarded as traumatic for the child sibling, potentially leading to enduring psychological and relational implications [23]. However, there is a paucity of research examining the extent to which changes in familial roles due to hospitalisation influence siblings, with only a limited number of studies addressing this topic to date [6,19,21,24,25,26]. In contrast, some researchers reported divergent findings, with siblings described as demonstrating resilience and maturity in response to having a sister or brother with a CHC [27].

Additional studies identified positive outcomes, including heightened levels of empathy, enhanced coping skills, and positive changes in family dynamics [19]. These findings offer a contrasting perspective, suggesting that the experience of having a sibling with a CHC can foster growth and development, affirming the importance of recognising the variability of sibling experiences within the context of chronic illness. A study by Hastings [28] identified sibling age and parental gender, stress and social support as factors impacting on how the family responds and adjusts to these difficult life circumstances. The study highlighted differences in how parental gender influenced stress in families, with mothers reporting higher levels of anxiety, linked to both the child’s behaviour and the father’s mental health. Hastings [28] suggested this was likely due to mothers’ greater involvement in caregiving and household management than fathers, who showed stress patterns less directly influenced by these factors. These findings indicate the importance of considering gender roles in designing support interventions for such families.

Supporting these findings, a study on siblings of children with cancer, investigating the correlation between psychological adjustment and emotional support, [29] revealed that siblings who received adequate social support exhibited lower levels of anxiety and depression. This discovery is noteworthy as it highlights the critical role of social support and connectedness in navigating such experiences. Similarly, a recent qualitative study [18], focused on caregivers’ perspectives of hospitalisation and familial separation, and its impact for child siblings. It was noted within this study that many siblings experienced instability in various aspects of their life which likely caused considerable stress. The findings placed emphasis on siblings’ restricted visitation rights, exacerbated further by the COVID19 pandemic. While it has been suggested that visitation is helpful for siblings [18], the research surrounding the effects of limited visitation by siblings is still ongoing, thus further supporting directions of this study.

The impact of child hospitalisation extends beyond the immediate family unit to encompass a broader network of extended family members, neighbours, and community organisations [30]. Extended caregivers, such as grandparents, aunts, and uncles, to name a few, provide essential emotional and practical support to caregivers and siblings during periods of hospitalisation [31]. They often assist with caregiving tasks with siblings and aid with transport or manage household responsibilities while caregivers are focused on the hospitalised child’s needs [31,32]. Research suggests that this familial support can reduce parental stress and promote resilience [33,34,35,36] alleviating the burden on parents. Importantly, a longitudinal study [37] found that decreasing social supports, over a one-year period, was related to an increased risk for anxiety and depression for caregivers, reiterating the significance of understanding child hospitalisation from a larger systemic perspective. In essence, there is a tendency for fathers to utilise fewer social supports in comparison to mothers [38], highlighting again the importance of gaining comprehensive understandings of differing familial perspectives.

### Theoretical Frameworks

Understanding the impact of child hospitalisation on the family system benefits from an integrated application of multiple theoretical perspectives. Attachment theory [39] examines how hospitalisation-related separation can disrupt caregiver–child bonds, influencing emotional well-being and family functioning. Such disruptions can spread throughout the family system, affecting siblings and extended caregivers. Siblings who take on caregiving responsibilities may be particularly vulnerable to developing insecure attachments over time [38,39]. While attachment theory addresses relational bonds, cognitive development theory [40] complements it by explaining how developmental stage and cognitive processes shape perceptions and coping strategies. Younger siblings may struggle to understand the reasons for separation, which can heighten their distress, whereas older children may develop varied coping mechanisms. These developmental differences can both influence and be influenced by attachment security, indicating a reciprocal relationship between the two frameworks. Systems theory [41] and Bronfenbrenner’s ecological model [42] broaden the lens further, situating attachment and cognitive processes within interconnected social environments. These theories offer a broader view of environmental influences such as education and healthcare systems, community resources, and cultural norms that influence experience.

These frameworks offer complementary pathways for understanding the hospitalisation experience: attachment theory clarifies the relational role, cognitive development theory accounts for individual variation in interpretation and adaptation, and systemic models locate these within a broader socio-cultural context. Integrating these well-established theoretical frameworks into the study strengthens this research by providing various and contrasting perspectives to hold. Through this integration, this study is positioned to examine how relational, developmental, and systemic factors converge to shape the experiences of main caregivers, extended family caregivers, and child siblings when a child with a CHC is hospitalised. This multi-theoretical foundation directly informs the study’s objectives: to explore a comprehensive understanding of familial impacts across varied CHCs and to inform the development of relevant interventions that can be applied at the local, regional, national, and global levels.

## 2. Study Objectives

This study was designed to enrich the existing literature by understanding the impact of child hospitalisation on the family system, informed by a triangulation approach (main caregivers, child siblings, extended caregivers). Through qualitative inquiry of varying familial perspectives, the research aimed to explore the lived experiences, challenges, and impacts of families who faced child hospitalisation. The study aims to offer valuable insights for healthcare professionals and policymakers by identifying the unique challenges that families face, incorporating various CHCs to support the external validity of the findings. The findings of this research hope to help shape policy development and service provision, leading to better outcomes for families.

The research questions are as follows:

1. What are the experiences of main caregivers, extended caregivers, and child siblings facing child hospitalisation?
2. How are these experiences similar and/or different?
3. What are the positive and negative impacts on the family system?
4. What is the specific impact on a child sibling’s daily life, communication, and relationships with family members and others?

## 3. Method

### 3.1. Research Design Overview

The study employs a qualitative approach to explore the impact of child hospitalisation on the family system, using framework analysis [43,44,45]. The Framework Method can be defined as a systematic approach designed to make sense of qualitative data by first, identifying patterns, similarities, and differences across the data set, followed by exploring how different elements of the data relate to one another. This involves mapping out connections between themes and examining the nuances within and across these themes [45]. Semi-structured interviews were conducted, guided by an interview framework developed through literature review and in partnership with a Patient and Public Involvement (PPI) panel. Interviews were recorded using Microsoft Teams application. The Framework Method was selected as the most appropriate form of analysis, due to its design for use within health research. This technique is distinguished by its structured, step-by-step approach to data synthesis from different groups of participants, allowing for in-depth and across-data exploration, meeting the study aim of using a triangulation approach towards participant interviews.

### 3.2. Study Participants

#### 3.2.1. Researcher Description

This research project constituted partial fulfilment for the Doctorate of Clinical Psychology degree programme. The research project was conducted by LM, a trainee Clinical Psychologist, under the supervision of qualified Clinical Psychologists and Professors affiliated with Queens University Belfast (QUB), Ulster University, and the Department of Health (DoH) within Northern Ireland. The research team has a keen interest in this area due to recognising gaps in the literature and clinical practices, influencing the innovative directions of this project. The main researcher (LM) maintained a reflexive journal throughout the interview process to enable the identification of any biases that could potentially influence the interpretation and analysis. In addition, the potential influence of the researcher’s clinical role was addressed by clarifying the research (non-clinical) context to participants, employing neutral and non-directive questioning, and refraining from offering clinical advice during interviews.

#### 3.2.2. Participants

Participants comprised eight primary caregivers (*n* = 8), all of whom identified as mothers, seven extended caregivers (*n* = 7), including fathers and grandparents, and eight child siblings (*n* = 8), aged between 4 and 16 years (Table 1). Participants all resided in Northern Ireland and met the eligibility criteria (Table 2) for the study. Participants were unknown to the researcher prior to participation.

#### 3.2.3. Patent and Public Involvement (PPI) Panel

Relevant PPI panels organised through local hospitals and QUB university were identified in adherence to the National Institute for Health research (NIHR) standards for PPI [46]. Participants who agreed to be contacted via PPI were invited to review the interview guide and research proposal, offering crucial feedback for refinement prior to commencing the study. Interested panel members were provided with details regarding the recruitment process, including eligibility criteria and informed consent procedures. The panel consisted of main caregivers, extended caregivers, and child siblings who offered their expertise and feedback, co-producing the design of the study. PPI panel members were compensated for their time and involvement [46].

### 3.3. Participant Recruitment

#### 3.3.1. Recruitment Process

Recruitment was conducted through the NI Children’s Health Coalition. A research statement was provided to the Director of the Coalition and presented to their Board of Directors for approval (Supplementary Material SA). Three advertisement bursts through the coalition’s social media platforms were agreed. A study advertisement (Supplementary Material SB) was disseminated through the coalition’s eleven charitable organisations’ social media accounts. The main researcher (LM)’s email address was provided on the study advertisement for interested participants to access further information regarding the study. This enabled the research to be advertised to a large audience with varying CHCs. After each bout of recruitment, interested participants who contacted LM were provided with a participant information sheet (Supplementary Material SC) and advised to respond to LM via email with their contact (telephone) details to begin the screening process. The research aimed to recruit 10 families. This decision was made collectively by the research team to ensure that the sample size is large enough to estimate the probability of individuals with similar characteristics sharing the opinions being represented [47].

#### 3.3.2. Participant Selection

Participants were selected following the recruitment process as above. During the screening process, participants answered questions asked by LM to determine their eligibility based on inclusion and exclusion criteria (Table 2). The study selected families for individual interviews, involving a main caregiver, a chosen extended caregiving member (any member of the family that supported in caregiving duties of the sibling(s), and a child sibling. To ensure meaningful participation, child siblings needed to be at least 4 years old, capable of engaging in the interview process, and able to communicate effectively. Participants who passed screening, provided their email addresses to receive consent and information forms, which were completed and returned via email before the interviews took place. Each recruitment phase targeted interested families, resulting in eight families being recruited overall.

## 4. Data Collection

Qualitative research has recognised the popularity of semi-structured interviews as a data collection method, attributing their versatility and flexibility [48]. The utilisation of semi-structured interviews in this study was deemed appropriate, given previous research highlighting their efficacy with both individual participants and groups [49]. Semi-structured interviews proved invaluable in this study, offering a flexible framework for inquiry while allowing for the exploration of unforeseen themes. This approach empowered the researcher to delve into participant responses naturally, prompting follow-up questions that yielded rich and nuanced data. Each family unit participated in three separate interviews, one with the main caregiver, one with the child sibling, and one with the extended caregiver. Individual interviews were conducted to promote confidentiality, safety, and an accurate depiction of each individual experience. Each family was asked to select their preference for interview, either using Microsoft Teams application (*n* = 7) or face-to-face (*n* = 1). Main caregivers’ interviews were held for 60 to 105 min, with an average interview time of 90 min. Child sibling interviews were held for 20 to 30 min, with an average of 20 min. Caregivers remained present during the child sibling’s interview if the child was under the age of 16 years, following ethics committee guidelines. Age-appropriate modifications included the use of simplified language, shorter question formats, and the incorporation of visual prompts to aid comprehension and engagement. The interview schedule was informed by the existing literature on qualitative interviewing with young children and was reviewed by clinical psychologists experienced in paediatric work to ensure developmental appropriateness. While a formal psychometric validation of the instrument was not conducted, the schedule was piloted with caregivers and children of similar age to refine clarity and relevance. An interview guide (Supplementary Material SG) consisting of adapted language to use based on participant age was meticulously followed. Extended caregivers’ interviews were held for 45 to 60 min, with an average of 45 min. Interviews were recorded using Microsoft Teams application and automatically uploaded to Microsoft Stream via QUB OneDrive secure system. Theoretical saturation was not used as a criterion for concluding data collection. This decision was based on the study’s exploratory and context-specific aims, which sought to capture a broad range of perspectives within a pre-defined participant group rather than achieve thematic exhaustiveness. Sample size was determined based on feasibility, ethical considerations, and the need to minimise participant burden, particularly for young children and their families during a potentially stressful time. A reflexive journal was maintained by the researcher LM to document initial impressions, ideas, and early interpretations of the data. This journal served as a valuable tool for developing awareness of researcher heuristics. Analytic memos were diligently recorded during the interview stage, serving as prompts for discussions on themes, concepts, or ideas within the research team and with participants, consistent with Framework analysis (Supplementary Material SH).

## 5. Analysis

The Framework Method for analysis was used, enabling themes to be developed inductively from the stories (experiences and views) of research participants. An initial framework matrix (Supplementary Material SH), developed in accordance with the Framework Method guidelines [45] served as the foundation for subsequent deductive analysis of the remaining transcripts. The analysis consisted of seven distinct stages. In the initial stage, the interview recordings were automatically transferred to a secure site via Microsoft Stream. Data was transcribed verbatim. In stage two, time was dedicated to familiarisation of the data, allowing for the recording of analytical notes and initial impressions. Stage three involved inductive coding independently by two researchers (LM and PA) for randomised transcripts of a main caregiver, an extended caregiver and a child sibling. Both researchers familiarised themselves with the data by reading the transcripts multiple times. LM and PA engaged in open coding independently and assigned preliminary codes. To assess the consistency between the two coders, intercoder reliability was evaluated using Cohen’s Kappa. The calculated reliability coefficient was 0.78, indicating substantial agreement. Any discrepancies in coding were identified and differences discussed. Through consensus meetings, both researchers resolved coding conflicts by refining code definitions, collapsing overlapping codes, or creating new codes where necessary. Stage four involved the full research team convening to discuss and agree upon a set of codes that could be universally applied to all subsequent transcripts. During stage five, the established framework was applied to index the remaining transcripts, systematically organising the data according to the identified codes and categories. To enhance clarity and facilitate exploration, the data was then charted onto a matrix within a Microsoft Word document, supported by colour coding to explicitly highlight codes and categories (Supplementary Material SH). In the final stage, further interpretation of the data involved discerning similarities and disparities across the data set, probing into potential relationships and patterns. During this stage, the similarities and differences between the data was identified to generate themes and interlink ideologies with the pre-existing literature.

## 6. Results

Overall, eight families participated in the research (Table 3). Prior to interview, one extended caregiver decided to withdraw their consent to participate, resulting in a total of 23 interviews being conducted. All main caregiver (MC) participants identified as mothers, with a mixture of fathers and grandparent participants as extended caregiving (EC) members. All sibling participants were child siblings (S), ranging in age from 4 to 16 years. The findings from the eight families are summarised into differing family member perspectives using codes (1–8). The main themes derived from the data include emotional impact, relational impact, practicalities, adjustment for the family system, communication and “take-home messages”, with subthemes for each category identified (Table 3).

### 6.1. Emotional Impact

Subthemes: traumatic impact of repeated hospitalisations; the unknowns; ongoing impacts.

#### 6.1.1. Traumatic Impact of Repeated Hospitalisations

##### Main Caregivers’ Perspective

One striking aspect of the data was the emotional implications experienced by participants, particularly main caregivers, due to the trauma associated with the hospital experience. The data consistently afforded insight into the caregiver’s experience of anxiety, trauma, depression, and post-traumatic stress (PTS) symptoms due to the nature of their child’s medical journey. Numerous caregivers vividly described the physical manifestations of their emotional distress, including symptoms of “nausea” (MC1), “sleep disturbances” (MC1,4,5,7), “panic attacks” (MC2,4) and “feeling jittery” (MC1). One caregiver expressed, “I’ll feel sick for days. I’ll have knots in my tummy and feel jittery” (MC1). Another caregiver vividly recounted, “my throat was closed, and I couldn’t breathe” (MC4). Main caregivers also articulated a range of emotions, from “fear” and “worry” to “irritability” and “low mood”. The emotional response acknowledged most by caregivers was the “traumatic” nature of the experience. Without receiving formal diagnoses of post-traumatic stress disorder (PTSD), caregivers simultaneously felt these effects: “I just wanted to survive and make sure everyone else around me was surviving” (MC1), “I would have a lot of post-traumatic stress around the hospitals now too, and do anything to avoid them” (MC1), “I think you do get PTSD, but you don’t get it straight away” (MC4), “I have nowhere near the same coping skills that I used to. I’m just on high alert. It just brings back so much trauma” (MC4). Caregivers described the enduring impact of their distress and its continuous impact on daily life. Notably, several caregivers reported their emotions can be triggered by routine activities such as “queuing for hospital car parks” and “attending outpatient appointments” (MC1,2,4,6), regardless of who the appointment is for: “Once you know the next one’s coming, I can just feel that anxiety start to rise again” (MC6). Others reported they find it difficult to watch TV programmes containing or related to hospitals, “I can’t watch TV shows if there is a hospital in it” (MC7) as this was identified as a trigger for emotions. One caregiver stated, “It takes a week after the hospital for my body to return to normal” (MC1). All caregivers described the importance of “holding onto hope”, regardless of whether the information relayed to them suggests otherwise: “I tried to ignore facts and go on anything hopeful” (MC1). They described the intense fear they experience during each hospital stay, facing uncertainties and fear of losing their child: “each time we are admitted, we wonder are we going to lose him this time” (MC1). Uncertainty and unpredictability emerge as contributing factors to the traumatic impact, permeating the underlying fear and worry that accompany each visit to the hospital: “There was always a lot of uncertainties” (MC1,2,8).

Another important factor identified in the data contributing to the traumatic impact was the duration and frequency of hospital admissions, “I stopped counting because I just couldn’t count anymore” (MC1,7), and the rarity of the condition: “No one knew what to expect with his condition, which made me more anxious” (MC1). Caregivers specifically referred to the Neonatal Intensive Care Unit (NICU) and the traumatic impact of witnessing medical interventions and being separated from their baby/child. One caregiver described the experience as “unbearable to think about” and “horrendous” (MC6). Another caregiver described the traumatic experience of witnessing their child be resuscitated on numerous occasions, “we watched him be resuscitated nine times … I don’t think we will ever get that out of our heads” (MC1). Being separated from their CHC child and watching their child be taken for surgery was prominent in contributing to the overall traumatic impact. One caregiver reported “watching your child be taken away from you was the scariest part” (MC3), “we didn’t get to see him again until that night … they took me to the maternity ward with all the mummies and babies without him, it was horrific” (MC1).

Feeling disconnected from emotional and physical states was evident for caregivers: “I had pneumonia and I didn’t know … I was completely disconnected from myself” (MC1).

Feelings of mental, emotional and physical exhaustion are evident, with one caregiver expressing concerns about their own mental health, with the fear of “cracking up” under the constant strain of fatigue: “I didn’t want to tell a soul how I was feeling cause I really thought that maybe I’m cracking up or something” (MC1). This fear reflects the immense emotional distress and psychological pressure that caregivers experience during their child’s hospitalisation. In addition, caregivers reveal that caring duties take a significant physical toll on them. One caregiver described their experience of weight loss due to neglecting their own nutritional needs amidst the stress and uncertainty of their child’s hospitalisation: “I had lost a lot of weight because my body was already under stress, I actually thought I was going to have a breakdown” (MC4).

The data shows a range of emotions experienced by the caregivers, including fear, worry, helplessness, guilt, isolation, overwhelm, and prolonged grief. These feelings were further exacerbated by the fact that caregivers can feel responsible for the care of their child, which can lead to additional stressors in trying to balance being a caregiver and a medical expert: “I had to learn the language” (MC7), “I felt like a secretary” (MC1). Caregiver guilt was expressed in many ways. For instance, following medical interventions for the CHC child, “it felt like we were torturing him” (MC1). Another common source of guilt was not spending enough time with siblings, as one caregiver expressed, “he went from being number one in the house, to the bottom of the pile” (MC4), and guilt of experiencing conflicting emotions towards their CHC child, as one caregiver reflected, “It’s awful but I did feel angry towards her [CHC child] and towards the situation [of hospitalisation and being separated from sibling]” (MC4).

Findings also afford insight into the emotional implications experienced by caregivers upon their child’s discharge from the hospital. For some caregivers, the emotional impact felt more prominent post hospital discharge: “When things slowed down, it really hit us” (MC1).

Despite receiving assurance from their child’s medical team, caregivers expressed apprehension and worry about transitioning their child back home, adding to the traumatic impact. The prospect of assuming sole responsibility for their child’s care after relying on medical equipment and expertise in hospital, created a sense of overwhelming fear and uncertainty:

It was really, really worrying taking him home. My whole family was saying, I’m sure you’re dying to get him home. And I was like, not a chance. He’s been surviving on all these machines. What am I gonna do when I take him home? So we were not dying to take him home. We were so nervous to take him home. (MC1)

The caregiver’s reluctance to bring their child home is palpable, contradicting the assumptions of other family members and friends who may perceive this as a joyous occasion. Instead, the caregivers expressed feelings of anxiety and trepidation, questioning their ability to meet their child’s needs outside the controlled environment of the hospital. This fear is compounded by the realisation that they will no longer have immediate access to medical support and guidance, amplifying their emotions. One caregiver highlighted the traumatic impact of being trained to administer medication and use machinery to aid resuscitation for their child upon discharge, stating “this is not something you do to a brand-new mummy … I was really angry and scared” (MC1). For some participants, the degree of traumatic impact varied depending on the hospital to which they were being admitted: “For me, the impact depends on which hospital I am going to. In one hospital, they are maybe more open to what I feel as a parent and this helps” (MC2).

Caregivers acknowledged their attempts to deny the severity of the situation and their child’s CHC, opting instead to focus on hopeful outcomes. This reflects a common coping strategy employed by individuals facing traumatic or challenging circumstances, wherein they seek comfort in positive thoughts and possibilities as a means of managing distress. Caregivers described being attuned to the traumatic and emotional impact on their CHC child. Some caregivers delved into the emotional and behavioural changes they notice in their child ahead of and during a hospital admission: “he would never settle … he would never sleep… he would be nervous of people coming to his bed and would ask alot of questions” (MC1). Another caregiver articulated, “She will cry constantly… She won’t go into hospital unless we are all with her” (MC3). One caregiver described the additional traumatic and emotional impact of being their child’s “emotional sponge,” (MC1), implying their role in holding and managing their child’s emotions, as well as their own and others in the family. This suggests a heavy sense of responsibility, where the caregiver acts as a source of emotional support and stability for their child during times of illness and distress. Many caregivers described the emotional toll of “standing by” and “watching” their child undergo medical interventions and articulated the intense emotional responses triggered by the medical language used during discussions about their child’s care. For instance, one caregiver recalled how the use of the word “Christened” (MC1) implied that her child’s condition was critical, potentially suggesting an impending loss:

It was awful because they had asked twice, did we want him christened? And to me this was a major threat, cause they weren’t just like, do you want to get him christened? They were definitely telling me that we were gonna lose him. (MC1)

This interpretation highlights the depth of emotional distress experienced by caregivers, offering valuable insight into the psychological and emotional ramifications of their child’s hospitalisation and condition. Mothers tended to experience more intense emotional impacts compared to other members of the family, “It was just awful, and I couldn’t understand why no one else felt like I did” (MC2), “I didn’t want to be positive like my husband “ (MC3), which can perhaps relate to being more present in the hospital environment with the CHC child.

##### Extended Caregivers’ Perspective

Fathers who participated in the research as extended caregiving members provided valuable perspectives that highlighted discernible distinctions regarding the traumatic and emotional implications of the same hospital experience. Numerous fathers indicated they were not in tune with their emotions, as they needed to “hold everyone together” (EC2,3,4,5,8), or “I didn’t have time to think about it” (EC1,2,4,8), “I needed to get on with it” (EC1,8). Other fathers shared that they felt “frightened” by the hospital experience, describing it as “scary” in their narratives: “The hospital is scary, you never know what will happen” (EC1). Despite these emotions, they also approached the situation from a different standpoint, recognising the hospital to be the best place for their child to receive care: “however I knew it was the best place to be” (EC1), “She had to be there, you just cope with it at the time, but I think we were a bit shell shocked” (EC3). Similarly to the main caregiver’s experience, extended caregiving members felt the most difficult part of the experience was watching their child be taken for surgery: “that’s the real tearjerker” (EC5). The sight of their child in pain or discomfort evoked more of an emotional response from fathers, with a sense of helplessness described, as they wished to protect and alleviate their child’s suffering and felt unable to do so: “I didn’t like that there was nothing I could do” (EC3), “she was in pain … it was so hard to watch” (EC2,4). One father shared the emotional impact of juggling care for his child with a CHC alongside his other children, highlighting the significant challenge it posed. He expressed, “it was very hard and tiring because you are dealing with the emotions of [child with CHC] being sick in hospital and [sibling] not being with us and missing them” (EC3). He further elaborated on the internal conflict, stating, “you never want to leave [CHC child], but also never want to leave [sibling] and make them feel left out” (EC3). This insight suggests a considerable emotional divide within the family system, making it difficult to strike a balance that considers the well-being of all family members.

Grandparent participants as extended caregiving members provided innovative insights into their perspectives of the emotional impacts within the family. One grandparent shed light into their own emotional implications from the hospital experience, describing the traumatic impact of walking into NICU:

I had no idea what to expect when I entered, and what I saw will always stay with me. I remember it so clearly, I can close my eyes and still see and hear it. It gave me the weirdest feeling, it will never leave me. (EC6)

This is indicative of the lasting effects the hospital experience can have and the distressing impact of the environment itself. Interestingly, the main emotional and traumatic implication described by grandparents was their concern for their own child (parent/caregiver) going through this process: “I was more worried about my own daughter” (EC6). Additionally, they described feelings of “inadequacy” and a strong desire to provide more extensive help and support: “I felt very inadequate and wished there was more I could do to help” (EC6). This reveals an emotional investment and sense of responsibility towards their own child’s well-being amidst the circumstances. Some extended caregivers felt there was little to no impact placed on the child siblings: “they seem to be fine with it, I don’t massively think it has affected them” (EC1). Meanwhile, others felt that the impact gradually became more evident when caring for the siblings during times of admission: “I’ve always noticed that after maybe three or four days there would be signs of anxiousness with the other children … Just maybe getting to sleep and maybe wee temper things” (EC6). Another extended caregiver gave similar reports: “it’s really when I look back on it that I realised they were getting anxious, I think they were thought to be coping better than they were” (EC2).

##### Child Siblings’ Perspective

The wave of emotions experienced by siblings was consistent with caregiving participants. Siblings reported feelings of “shock, worry, fear, anxiety, anger, confusion and sadness”. Some siblings reported feeling “nervous” (S3,6,7) during the periods of admission due to missing their sibling and being separated from their caregivers. Other siblings reported “liking” when their sibling is admitted to hospital due to being able to spend time with other family members completing enjoyable activities: “I like it because I get to go to grannies and play” (S5). One sibling reported frustration with admissions: “I don’t like when my plans change or things get cancelled … it’s annoying” (S6). Others reported feeling “sad” as they “do not like it when their brother/ sister is in pain” (S7,8). Attributed to the traumatic impact, many siblings shared vivid memories of the hospital experience, including memories of ambulances, doctors’ and nurses’ uniforms, and specific colours that stood out to them: “They were wearing white” (S4). Siblings reported developing negative associations with hospitals, “hospitals are now scary to me too, I don’t like them” (S3,6,7,8). Other siblings shared vivid details and memories of when their sibling with a CHC was admitted to hospital and reported “it’s not something I will forget” (S6,7). The emotional implications for siblings were evident within the data, with descriptions suggesting the large impact the hospital experience had on them also.

#### 6.1.2. The Unknowns

Subthemes: Fear of the unknowns and the uncertainty and unpredictability of the hospital experience were a prominent theme occurring for all participants, which exacerbated the emotional impacts experienced.

##### Main Caregivers’ Perspective

Caregivers expressed feeling threatened by the unknowns, reflecting a pervasive fear of losing their child. They reported experiencing frequent and various unknowns stemming from diagnosis of their child’s CHC and subsequent hospitalisations. Caregivers reflected on the various questions they battled with when their child was hospitalised: “how long will this stay be?” (MC2,3,8), “what is going to happen?” (MC4,5,6,8), “are we going to have to come back in again when we get home?” (MC1,7). Caregivers described the enduring emotional implications of these thoughts, manifesting as worries and fears. The ongoing nature of these worries exacerbated emotions prior to upcoming appointments or admissions and triggered further worries of their child’s future and future medical journey, posing questions like “what is his life going to be like?”, “will he receive the right support?”, “who will look after him in future?” (MC1,5). Other caregivers expressed fears of the unpredictable nature of their child’s condition, “no one knows what’s going to happen” (MC1), leaving them feeling constantly on edge. Due the uncertainty and unpredictability of hospital admissions, one caregiver discussed the difficulties surrounding daily life and future planning amid the unknowns, “It is difficult to plan anything, especially holidays as we never know what is going to happen with (CHC child’s) health” (MC2,6).

##### Extended Caregivers’ Perspective

Extended caregivers also shared this experience: “I don’t know what our future looks like” (EC5), “We don’t know what is going to happen” (MC2,3). Others reported that forward planning is a challenge and discussed its implications for the family system: “We can’t book any holidays, this is hard on everyone” (EC3), “It’s hard to plan ahead” (EC1,5), I sometimes don’t know if I should just plan stuff for the other kids or not“ (EC3). This affords insight into the emotional implications that can arise due to uncertainties and unknowns about the future. In addition, the data posits concern for the “other kids [siblings]”, and the potential impact of the unknowns on their daily life. Amidst the unpredictability, fathers adopted a more practical response to “the unknowns”, compared to mothers. They reported feeling the need to “keep things running at home” (EC2,5) for the family.

##### Child Siblings’ Perspective

For siblings, the hospital experience is charged with confusion and anxiety stemming from the lack of understanding about their sibling’s condition, treatment, and hospital admissions. Some articulated their distress, expressing how the uncertainty of the situation amplifies their worry: “If don’t know who is going to hospital? who is staying? where am I staying? I worry more” (S7). Numerous siblings reported the most difficult part of the hospital experience is “not knowing or understanding” (S7), “feeling confused” (S6), and “wondering” (S6) about the well-being of their CHC sibling. In support of this finding, other siblings reported finding the hospital experience “better” when plans were in place and communicated to them: “It’s better when I understand it more” (S7). Siblings felt when their understanding of the process increased, they were able to cope better with the uncertainty and unpredictability of admissions, “If I know what is happening, I am ok” (S6,7).

Lack of communication regarding the unknowns appeared to increase emotional implications for the siblings: “when I do not know what is happening, I find myself wondering more …I become more anxious wondering what is happening and trying to piece a picture together in my head” (S7). Some siblings felt it was best to manage their own uncertainties and expressed wondering as to whether or not they should ask about their sibling’s health condition to their caregivers or the sibling themselves, “as I’ve gotten older, I think I have more questions about it, but I don’t know whether to ask them or not” (S7). The data posits that siblings longed for more clarity, communication, and reassurance amongst the unknowns. Some siblings expressed curiosity into why their sibling has the condition and wondered what would have happened if they had the CHC instead. One sibling reflected, “I always think, what if that happened to me [twin sibling] (S7)”. Notably, siblings appear to express a heightened desire for knowledge, yet they also grapple with the fear of disrupting the delicate balance within their family dynamic. Consequently, they often find themselves sitting with unanswered questions, “It’s just a bit confusing because sometimes I don’t know what’s going on” (S7). Siblings who were attuned to their caregiver’s emotional responses anticipated their sibling’s hospital admissions based on subtle cues: “I often know when (CHC child) has to go to hospital because mum and dad are stressed” (S7). This suggests that some siblings may use cues to interpret information that is not being communicated directly to them.

#### 6.1.3. Ongoing Impacts

##### Caregivers’ Perspective

Caregivers reported facing continuous emotional and traumatic challenges during outpatient appointments, follow-up visits, and with ongoing hospital admissions. Nearly all caregivers reported wanting to avoid hospitals and had developed negative associations with hospitals, “I would do nearly anything to avoid going to hospitals” (MC4), “When we have appointments, we don’t want to go, it brings out the worst in us all” (MC1). The constant exposure to medical settings triggers heightened emotions and stress, impacting daily lives and family dynamics. Caregivers reported that their CHC child endured ongoing impacts stemming from their hospital experiences, manifesting in various aspects of their lives. The overspill of their hospital experiences impacts their social, educational, and daily life domains, posing challenges to their development and overall well-being. Sensory implications for the CHC child have been noted by caregivers, with numerous reports suggesting the hospital experience contributed to heightened sensitivities to noise and food textures, continuing to impact sleep and eating habits at home. One caregiver reported that feeding became a huge issue at home: “Feeding became a huge issue as he was very oral adverse because of the negative things with tubes and wires and different operations” (MC1). In addition, all caregivers shed light into the educational and relational difficulties their CHC child now experiences: “he has different needs and requires support in all areas” (MC1), “they need extra support in school” (MC3,6,7,8), “he struggles to make friends as they don’t understand his needs” (MC1,5).

##### Child Siblings’ Perspective

For siblings, continuous exposure to hospitals and the uncertainty surrounding their siblings’ health can leave lasting effects. Siblings reported developing hypervigilance and feeling “on edge all the time” (S6,7), anticipating the next medical emergency. Hospitals become familiar yet distressing environments for siblings: “I hate hospitals now too” (S3,6,7,8). The frequent visits to the hospital disrupt their sense of security and stability, as they witness their siblings’ pain and suffering. The “sight of medical equipment” (S7), the “sounds of machines” (S7), and the “smell of antiseptics” (S8) are reported to evoke emotions. Difficulties in peer relations were reported as a major ongoing impact for siblings. Siblings felt they struggled to relate to their peers who may not fully understand the challenges they face, exacerbating feelings of isolation and feeling different: “My friends don’t even want to go into my house, they don’t want to make [CHC child] sick or anything” (S7); “I normally go to their house, mum doesn’t like people being in the house in case they are sick” (S6).

They report their social activities and ability to maintain friendships is challenging due to the demands and unpredictable nature of their siblings CHC: “I don’t get out to see them [friends] as much because I am not motivated to go” (S6), “It’s hard to see them, like its mum and dad swapping over every night and then me in the middle of homework and then its oh, lets go to the hospital now“ (S7). The associated emotional distress can largely impact academic performance. Some siblings faced significant academic challenges, prompting them to seek additional support in school, and others recounted memories of behavioural struggles, prompting assessments for conditions such as attention deficit hyperactivity disorder (ADHD) and autism: “It had a massive impact on me in school, my behaviour wasn’t great and then I got diagnosed with ADHD” (S8), “I remember finding it hard to focus on my work“ (S6), “I had to get help from the classroom assistant when I was younger“ (S7), “I am waiting on assessments in school” (S6). These assessments remain ongoing for some siblings, and some siblings younger in age are currently awaiting assessments, as reported by their caregivers. This reflects the complex and ongoing nature of siblings’ educational and developmental journey. Some siblings report feeling “lonely” and “sad” (S1,2,3,6,7) when thinking about the ongoing impact the hospital experience has on them. The unpredictability of their sibling’s health may overshadow their own aspirations and goals, leading to feelings of resentment and hopelessness, with one sibling reporting, “I find it hard to focus on my own needs” (S6), suggesting further continuous impacts.

### 6.2. Relational Impact

Subthemes: bonding in the hospital setting, relational disrupters, relational maintainers.

#### 6.2.1. Bonding in the Hospital Setting

##### Caregivers’ Perspective

The relational impact of hospitalisation was particularly evident in the data for caregivers whose children received a CHC diagnosis in utero or after birth. Several caregivers shared the emotional distress they experienced upon being offered the option of abortion and the lasting impact this had on their perceptions and expectations regarding their child: “When we found out our child had (a CHC) when pregnant, we were offered an abortion” (MC1,5,7), “I was taken aback by this” (MC1), “I didn’t know how to feel, or how to feel towards my baby” (MC5), “We chose not to and we lived by that choice” (MC5). This significant moment shaped their perspectives on what their child’s life would entail, influencing their outlook and emotional connection to their child from early on. In contrast, some caregivers expressed a different viewpoint, highlighting that having the option of abortion was significant for them. They emphasised how this choice heightened their capacity to nurture feelings of love and care for their unborn child, feeling empowered by their choice and ability to connect with their child after birth: “I just kept thinking, what if we didn’t have our little angel” (MC1).

Caregivers, particularly new mothers, highlighted the importance of expressing milk and feeding their baby whilst in hospital to aid in the bonding process. One caregiver reported expressing milk for her baby was a positive memory she held about her experience of hospitalisation: “If I was awake and able to express, I would go and give him his fresh milk and they would tube feed him it … it was really nice and made me feel more bonded (MC1)”. Similarly, other caregivers outlined the significance of being involved in the process of administering medicines to their child/baby: “I wanted to be the one to give medicine to my baby” (MC1,4,5), and being able to care for and respond to their needs, “It was important that I changed him and cared for him” (MC1). A recurring theme among caregivers was their depiction of the supportive nature of hospital staff in promoting rest and aiding with feeding: “The nurses always tried to make things easier“ (MC4), “They would offer to feed him” (MC1). While some caregivers found this assistance beneficial, others experienced it as a hindrance to the bonding process, evidenced by phrases such as “It was important for me to do it … (MC1)”, “I wanted to be the one …” (MC1,4,5), which is an important understanding for healthcare staff in practice.

Dependent on the child’s CHC, caregivers report disruptions of frequently moving hospitals and the impact this placed on bonding with their children, other families, and medical teams: “you really get to know the families and nurses that work there, and then you’re uprooted and shipped out again” (MC1,5). This indicates that bonding and sharing this experience with others in similar circumstances was important for caregivers and had a positive relational impact. Some caregivers also reported challenges in leaving their child in hospital and in the medical team’s care: “I found it hard to leave him with others to look after him” (MC1). They reported feelings of fear and worry that their child would not develop trust in them as a caregiver, if they were not present with them in hospital for a short length of time: “that was the first time I’d ever left his side and wasn’t there to protect him … it wasn’t ideal being moved around so much” (MC1). Constantly being transferred hospitals was a key experience for numerous caregivers, who reported that they themselves struggled to bond with staff and suggested that bonding with the medical team is a contributing factor to a more positive hospital experience: “knowing the staff and the teams made my experience easier… there’s a genuine love for [ CHC child] … there’s a genuine care. I suppose that’s the bit that I get strength from” (MC7). One caregiver shared their experience of “co-sleeping” with their child as a deliberate strategy to foster attachment and strengthen their bond. This bonding strategy was described as a reciprocal process, with the caregiver expressing, “she needed me, and I needed her” (MC6). This mutual need for closeness affirms the emotional connection between the caregiver and their child, highlighting the importance of nurturing relationships within and beyond the hospital setting.

##### Extended Caregivers’ Perspective

Extended caregivers actively facilitated bonding for siblings: “I drove the children to the hospital at least once a week” (EC5), “we used facetime to make sure they kept in contact” (EC3,5,6,8), “we made sure to facilitate them playing together” (EC2,3,4). They utilised technology to ensure they remained connected, bridging the physical gap imposed by the hospital setting. They placed emphasis on engaging in play, utilising play therapists, and engaging with activities in the hospital setting. This appeared to be important in uplifting spirits of all family members, creating a positive bonding environment.

#### 6.2.2. Relational Disrupters

##### Main Caregivers’ Perspective

The theme of social isolation was prevalent for caregivers and their child with a CHC, as few relationships outside of the immediate family were reported: “they only really have us” (MC 1,5,8). Caregivers felt their child may be lonely as they spend significant amounts of time in hospital, away from educational and social settings. Due to spending so much time in hospital, the relationships formed between the caregivers, the CHC child, and the medical teams were identified as extremely important: “the hospital staff became like family” (MC7). From the caregiver’s perspective, the CHC child experiences many negative interactions in hospital settings, which contributes to strained relationships between the child, their caregivers, and medical professionals: “people [hospital staff] were constantly having negative interactions with him and he couldn’t understand that it was to help him” (MC1). These negative interactions may be perceived as threats by the child, exacerbating fears and distress regarding relationships, thus undermining their trust in caregivers and ability to feel safe and secure in the hospital environment.

The inability to fully comprehend the child’s hospital experience can exacerbate caregiver distress, as they perceive a breakdown in trust and connection between themselves and their child, leading to relational disruptions: “he [CHC child] didn’t want to be comforted by me” (MC1). In addition, numerous caregivers report notable disruptions in relationships between themselves and their other child/ children, who they were separated from for significant periods of time: “our bond [sibling and caregiver] was broken when I had to prioritise staying with… [CHC child]” (MC4). These relational disrupters appear to manifest in different ways, with another caregiver reporting, “they now seek out their dad when they are hurt” (MC5); “he [child sibling] does not look for me when I come home” (MC7). Another caregiver (mother) described the importance of the relational bonds formed between the child sibling and extended caregiver (father) during periods of hospitalisation: “they became so close” (MC5); however, she also recognises the attachment between herself and child sibling at home has undergone strain and alteration: “he doesn’t seek me anymore” (MC5). Due to siblings being required to stay with relatives or neighbours during hospitalisation, there were time periods where caregivers had no contact with them, “I had two other young children at home who I did not see for weeks” (MC5), highlighting the extent of familial separation.

Another caregiver described incidences of separation anxiety noticeable from siblings: “he would become so upset and wonder when I am coming home” (MC4), “they would be really clingy and cry going to school” (MC3). Caregivers reflected on the emotional and relational impact of their absence on the child sibling: “I didn’t even realise how neglecting I was towards … [child sibling]” (MC 4,8), “It was the loss of me that was her biggest anxiety” (MC7), “he [child sibling] was too young to understand… he must of been thinking mum’s left me” (MC4). Another caregiver acknowledges the presence of jealousy and resentment towards them, quoting, “there is a lot of jealousy and resentment towards me that I wasn’t there” (MC6). The ramifications of this imbalance extend to some sibling’s constant need for attention, as expressed by one caregiver: “he constantly seeks me and my attention” (MC4). Interestingly, caregivers also reflected on the disparity in attention and concern received by the child siblings compared to their CHC child and expressed frustrations regarding this: “everyone asks about [CHC child], and no one asks about [child sibling] at home… I found this hard” (MC4); “ you know I also have a child at home and I think people [staff] forget that” (MC8). This echoes the frustrations surrounding the lack of recognition and support for the sibling’s relational needs.

Caregivers also point to disruptions within their own peer relationships due to the process of child hospitalisation. One caregiver described the peer dynamics that occurred after their child’s diagnosis: “those first few years were probably the loneliest because it’s not that I didn’t have friends, but they didn’t know what you were going through” (MC4). This sheds light on the difficulty in finding understanding and support from peers who may not comprehend the complexities of the situation, resulting in disruptions within relationships. Pride and reluctance to ask for help also emerged as a common thread amongst caregivers, ”we didn’t want to ask others for help… it was a pride thing” (MC1), potentially impacting their relationships with others and further intensifying feelings of isolation and self-reliance. A recurring theme was the significant strain on their relationships with each other. One caregiver expresses, “we were like ships passing in the night for years” (MC1), and another caregiver describes the experience of “not seeing or spending time with each other” (MC2). This encapsulates the sense of disconnection experienced by caregivers as they become consumed by the daily routines and challenges associated with the hospitalisation. The focus on the CHC child’s needs reportedly dominates conversations and interactions between the caregivers, leaving little room for meaningful conversations that are not hospital-related. As articulated by one caregiver, “even when we are together, it’s always just talk about [CHC child] and his appointments” (MC1). This constant preoccupation with caregiving duties limits opportunities for parents to connect on a deeper level, perpetuating feelings of emotional distance. Caregivers reported feeling overwhelmed with caregiving responsibilities, leaving little time or energy for nurturing their relationship with each other. As one parent reflected, “any conversations we had was basically catching up on notes, we had other priorities” (MC1). This signifies the changeable dynamics within parental relationships, as they prioritise their CHC child’s needs over their own emotional connection. It is important to note that some caregiver participants reported to be separated or divorced, “It placed a massive strain on our relationship” (MC3), “we just weren’t there for each other” (MC2), indicating the significant relational implications that can occur as a result.

##### Child Siblings’ Perspective

These findings are consistent with reports from siblings, wherein feelings of anger and resentment towards their CHC sibling and caregivers were prominent, particularly during times of separation and if siblings were not fully informed or understood why they were separated from their family. They reported disruptions in relationships with their CHC sibling: “It was nice not having [CHC sibling] at my grandparents”, “she gets all the attention and it’s annoying” (S6). Caregivers feel that the strained sibling–sibling relationship is evident throughout the family dynamics, with one caregiver reporting that her CHC child feels that her sibling “doesn’t like her” and “becomes impatient with her” (MC4). Siblings also reported relational implications within their peer relationships. A common experience amongst siblings that arose in the data is the lack of peers allowed into the family home due to risks of infection or illness. One sibling reported, “my friends don’t want to come into my house in case they make her [CHC child] sick” (S7), which contributes to relationship divides. Other siblings report socially withdrawing from their peers due to a lack of motivation to socialise: “I’m not as motivated to go meet them” (S7), “there is always something going on at the house or mum and dad are now swapping shifts at the hospital and I want to be able to see them” (8). Siblings collectively reported feeling isolated or lonely, “I feel isolated, it’s just me and my family” (S3,6,7,8), and “feeling sad that I couldn’t see or talk to my friends when [CHC sibling] was in hospital” (S3,4,6,7). This showcases the relational implications that can occur from a child sibling’s perspective. Siblings differed from caregivers in this respect as they reported developing relationships with peers through schooling; however, they also reported that the hospital experience disrupts their ability to consistently engage in social activities with their peers.

#### 6.2.3. Relational Maintainers

Viewed from diverse familial perspectives, relationship maintainers emerge as a central theme, highlighting the important role they play in preserving familial bonds during periods of hospitalisation.

##### Main Caregivers’ Perspective

Caregivers provided insight into the overwhelming nature of their duties, expressing gratitude for the presence of having another person or an extended caregiver present with them: “I would have been lost without my sister there” (MC4), “if it wasn’t for my parents, I don’t know what I would have done” (MC2,5,6,7,8). The importance of maintaining relationships emerges as crucial for children with a CHC and their caregivers in the hospital setting. This experience is reflected through caregivers’ recognition of extended caregivers’ contribution and the impact this had on strengthening and maintaining familial relationships. This contribution stemmed from completing practical tasks, caregiving duties, or providing emotional support. This appeared to be valuable to main caregivers, as one caregiver reflected that “having huge help from family members made all the difference” (MC2). This collective effort reinforces the importance of shared experiences in maintaining relations within the family unit. In addition, access to and support received from other resources such as friends, charities, and organisations was also highlighted in the data as a contributor for relational maintainers. Many caregivers sought support from organisations and reflected on the importance of meeting “like-minded” families and families who have “shared similar experiences”. One caregiver described the friendships she made with other mothers: “I met other mums who have been through this, and just listening to them and how strong they were, helped me massively” (MC1).

##### Extended Caregivers’ Perspective

A father’s sentiment, “It was nice to sit there and have a cuddle” (EC3), signifies the importance of nurturing connections with the CHC child for themselves and caregivers.

The experience shared by grandparents illuminated a sense of gratitude for the opportunity to be present and offer support. One grandparent reflected, “I was grateful that I was able to be there and help” (EC6). Yet, alongside this gratitude, feelings of inadequacy arose as grandparents reported they longed to do more to help: “I just felt very inadequate” (EC6). The experience shared by one grandparent, “the hardest part was watching my own daughter go through this…I wanted to be there to help her” (EC6), highlights the emphasis placed on nurturing the relationship with their own child by offering as much support as possible in attempts to alleviate some of the burden for his daughter.

##### Child Siblings’ Perspective

For siblings, the experience is multifaceted. While there may be feelings of happiness and contentment in the care provided by grandparents or other family members, there is also a sense of longing for parental presence: “It was nice going to my grannies house, but I missed mummy loads” (S7), “When I was younger, I used to miss mummy” (S6). Despite this, the bonds formed during these periods of care, characterised by shared experiences, fun, and creating memories, appeared to be important buffers in maintaining secure relationships. Siblings shared moments of connection: “I loved watching movies and having midnight feasts with my grandparents” (S6), “they [grandparents] spoiled us and loved to play with us” (S5), “it felt the attention was on me when I was with my auntie” (S8). Another sibling shared positive experiences and expressed gratitude for the care provided by their granny and auntie, affirming, “It was good… I was really happy being there” (S7).

Similarly to main caregivers, access to services and organisations also arose as an important aspect for sibling relationships, with many siblings describing experiences of meeting other children who also have a sibling with a CHC: “I met other kids that were the same as me, we played and it was really nice… they understood me better” (S6,7).

### 6.3. Practicalities

This comprises the following codes: finance and employment implications, environmental implications, service implications, practical benefits.

#### 6.3.1. Finance and Employment Implications

##### Main Caregivers’ Perspective

The financial strain of caring for a child with medical needs was a major concern for many caregivers, who frequently find themselves in a difficult position when it comes to balancing their caregiving responsibilities with work obligations. The demands of hospital appointments and admissions can make it challenging for caregivers to maintain steady employment, often resulting in job loss or financial difficulties as reported by caregivers. Many caregivers report taking “unpaid leave or sick leave repeatedly” (MC1,2,4,5,6,7,8) to attend to their child’s medical needs, which can further exacerbate their financial difficulties. Main caregivers faced significant challenges with employment, with some caregivers needing to opt out or change their employment contracts, “I had to give up my job, I couldn’t manage both (MC1,4), “I had to reduce my hours” (MC2,3,6), to care for their CHC child, leading to a reduction in household income. Another caregiver shared their difficult experience of being “let go” from their position due to not being able to meet the demands of the workplace alongside caring for their child: “l was told I couldn’t meet the requirements of the job anymore and they let me go” (MC8). Consequently, identity changes for main caregivers arose. One caregiver reflected:

I’ve always said the thing that keeps me going has been my work. My mental health and purpose relies on it … that’s what keeps me going. Then when I go to work, I can start to think about other things. (MC7)

Further challenges identified included the reluctance to seek financial support such as carers allowance in fear of not qualifying or understanding the processes for application: “l wish someone could have told me where to begin with applying for help” (MC1). Some caregivers sold their possessions or re-mortgaged their homes to support them in their financial difficulty: “we were lucky we had houses to sell, otherwise I don’t’ know what we would have done” (MC7). In addition, other financial challenges, such as the hidden costs of having a child in hospital, were evident throughout the data. Hidden costs such as purchasing food and clothes during hospitalisation or petrol and parking fees were reported to have a large impact on finances: “we were travelling up and down daily, and had to take two cars because we were taking it in slots” (MC1). Some caregivers described the costs of purchasing equipment for their home and frequently replacing clothing due to hospital emergencies: “when he would be admitted, they would tear his clothes off” (MC1). Similarly, a caregiver described the hidden financial costs of following medical advice: “we were told to boil his clothes before he could wear them …the clothes would shrink, and we would have to continuously buy new ones” (MC1). For other caregivers, the biggest financial impact was due to physical relocation and the requirement to go overseas to England for treatment. The expense of relocation, enrolling siblings into education, and buying new uniforms and furniture were some examples provided which put a strain on finances: “the cost of relocating and setting up our new life was unexpected“ (MC3). Caregivers also face the task of managing shared responsibilities while ensuring that their child receives the best possible care. This can lead to increased stress and anxiety, as caregivers struggle to balance their caregiving duties with work obligations and financial concerns.

##### Extended Caregiver’s Perspective

Extended caregivers, such as fathers, appeared to assume the role of financial providers for numerous families. Fathers reported they continued to work to ensure the family could “stay afloat” (EC1,3,5) and described organising their work routine around the hospital schedule, “My boss was flexible which helped loads… I was able to work around the hospital and school routine” (EC3). Some extended caregivers report that being self-employed was helpful in this circumstance, whilst others were required to move jobs for more flexible working hours: “I had to take another job with different shifts” (MC8). One father highlighted the busyness of his daily routine: “It was work, home to change and a bite to eat, over to the hospital and repeat” (EC1).

#### 6.3.2. Environmental Implications

##### Main Caregivers’ Perspective

The physical environment of hospitals presented a unique set of difficulties that can make it difficult for caregivers to provide the best possible care. One of the biggest challenges that caregivers face is finding facilities and amenities that are conducive to their comfort and well-being. This includes everything from comfortable chairs and adequate lighting to easily accessible restrooms, access to showers and beds, tea/coffee facilities, and reliable Wi-Fi. The noise of medical equipment, described as “relentless beeping and buzzing of machines” (MC1), can make it difficult for caregivers and their child to get much-needed rest, which can lead to exhaustion and burnout. One caregiver expressed concern about the implications of this noise on everyone’s ability to sleep and the subsequent impacts of this on well-being: “no one could get any sleep and we had a very grumpy little boy” (MC1). The basic needs of caregivers may only be partially met during periods of hospitalisation, exacerbating emotional implications and the ability to cope with the experience. Another issue that caregivers often face is the constant transferring between hospitals. Moving from one hospital to another can be incredibly disruptive to routines and can create a sense of upheaval. Depending on the location of the hospital they have been transferred to, other practical implications of increased travelling, decreased amount of time spent with CHC child, and disruptions within work and educational environments for siblings were noted: “we had to physically relocate… [sibling] had to start a new school” (MC3). Despite a recognition of the clinical efficiency of healthcare professionals, the hospital environment is reported to be a difficult and emotionally charged place that serves as reminders of their child’s condition: “It was always really busy” (MC1), “It was just filled with sick children and it was heartbreaking” (MC2). Caregivers reported noticing their heighted emotions more when in hospital and would often need to “get some fresh air” or “take a walk“ (MC1,2,4,6) to take a break from the environment.

#### 6.3.3. Service Implications

##### Main Caregivers’ Perspective

Access to support services is often limited, which can leave caregivers feeling overwhelmed, isolated, and struggling to cope. The challenge is exacerbated by underfunded NHS systems, lack of staff, and lack of information and supports available, which can make navigating the complex hospital and post-hospital care system even more difficult for families. Not only do caregivers face these struggles, but siblings and other family members feel this impact. One caregiver expressed, “there is limited support available for services, and this meant I haven’t been able to get a social worker” (MC1). This lack of support can make it difficult for caregivers to manage their own well-being and that of their families. Caregivers demonstrate a strong advocacy role in towards their child’s needs, as evidenced by their efforts in “reaching and overcoming barriers in services” (MC1,6,7). However, these efforts appeared to come with added responsibilities, with one caregiver likening their role to that of a “secretary” (MC1), yet despite their dedication, they express feeling let down and unsupported, stating, “we were very, very badly let down by lots of services” (MC1). This disappointment extends to their perception of the medical team’s approach, with caregivers expressing frustration at the perceived lack of proactive intervention and support, as one caregiver articulated, “we really should have been allocated someone and given that equipment from when he came home from neonatal” (MC1). Caregivers afforded insight into the multitude of tasks involved in caring for a CHC child, including keeping track of phone calls and managing interactions with various healthcare professionals: “My phone was never out of my hand in case someone rang about [CHC child]” (MC7). This speaks to the logistical complexities of navigating the healthcare system while simultaneously attending to the needs of their child. The quote, “it’s too much to do, looking after your child and all the phone calls” (MC7), conveys the sense of burden and exhaustion experienced.

#### 6.3.4. Practical Benefits

Amid the difficulties, pockets of practical benefits that offered respite and support for caregivers were highlighted as important aspects of their hospital experience.

##### Main Caregivers’ Perspective

Despite the financial strain and job loss experienced by many caregivers, some found “relief” in the reduced burden of attending numerous outpatient appointments and having their child’s medical team within reach. One caregiver shared that some aspects of the hospital environment, such as “ward rounds” and having medical specialists near and available, were very helpful for their child’s care and were a practical benefit of hospitalisation: “Different specialists were able to come and visit him, instead of us having to bring him up so it was great” (MC1). For other families, the hospital became a space where they could spend quality one-on-one time with their CHC child, away from distractions and the busyness of other parts of life: “I did love going into hospital with her sometimes… it meant I could forget about housework and other things that I needed to do” (MC2). Numerous caregivers described cherishing this quality time with their CHC child, away from the practical burdens of “cleaning”, “cooking”, and “looking after the family home” (MC2,3,4,6). Another caregiver found time for self-reflection, “spending this time in hospital was a wake-up call … I realised we can’t keep going as is. We need to look after ourselves better” (MC1), indicating increased self-awareness regarding the mental and physical difficulties they were facing. Moreover, caregivers found practical benefits in the opportunity to learn about their child’s medical procedures and spend time creating memory books with them: “we focused on doing nice memory books together” (MC3). These activities enhanced their caregiving skills and allowed them to preserve quality time.

##### Extended Caregiver’s Perspective

From a father’s perspective, their CHC child being an only child at the time of hospitalisation was considered a practical benefit: “he was our first child, so we didn’t have to worry about leaving [sibling] at home or leaving them with somebody else for those first few years” (EC1). This is an important consideration for the research due to the recognition of the potential impact on families who have more than one child.

### 6.4. Adjustment for the Family System

#### 6.4.1. Main Caregivers’ Perspective

For caregivers, adjusting to the family’s health circumstances with more than one child arose as a prominent theme. Main caregivers reported finding themselves at the forefront of caregiving duties. They described the difficulties with “juggling hospital stays”, “managing household responsibilities”, and “caring” duties for all their children (MC2,3,4,5,6,7,8). The adjustment for mothers can be challenging, as data indicates they feel overwhelmed by demands and face struggles in balancing their own self-care with the needs of their child with a CHC, often neglecting their own physical and emotional well-being in the process: “my priorities were my children” (MC2,4,5,6). Caregivers described experiences of identity shifts, as they not only become medical experts responsible for their child’s health; they also continue to fulfil the caregiver role for other siblings at home: “I had to learn everything about my child’s care” (MC7), “I tried to have time with [sibling] where we talked about anything not related to hospital or her sister” (MC6). Further adjustments of hospitals and appointments becoming “the new normal” was identified by all caregivers. One caregiver reflected, “this is our new life and we had to get used to it” (MC7). The loss of identity felt from giving up employment was also indicative for mothers: “my daily routine and day to day changed drastically” (MC4,6), “I didn’t really know who I was when all of this [hospitalisations] settled“ (MC4).

#### 6.4.2. Extended Caregivers’ Perspective

Extended caregivers, including fathers and grandparents, report that adjusting to the emotional toll of having a CHC child who is experiencing hospitalisations was particularly challenging, as they felt pressure to “remain calm” and “hold everyone together” in the face of emotional stress. They experienced feelings of “helplessness” and “frustration”, especially when unable to spend as much time in hospital compared to the main caregiver due to work commitments and providing emotional and financial support for the family: “I would have felt guilty not being there with [CHC child]” (EC5). Learning to be absent from the hospital environment and their CHC child was a significant adjustment period, with one extended caregiver stating, “it was a shock to the system” (EC3). Specifically for grandparents, navigating the balance between offering support and respecting caregiver’s decisions over healthcare was highlighted as a difficult adjustment: “I had to learn to know when to butt in and when not to“ (EC6). Grandparents focused on wanting to protect their own children from difficult decision making and grappled with the emotional conflicts of this experience: “it was difficult watching what this was doing to my own daughter” (EC6). In addition, grandparents found themselves acknowledging their own mortality and fears about the future, as they witnessed the impact of illness through their grandchild: “I saw the fragility of life … it made me realise that I am maybe not grateful enough to be in good health” (EC6). This perhaps created an additional layer of adjustment within the family system.

#### 6.4.3. Child Sibling’s Perspective

Siblings reported adjusting to family life with a CHC child was challenging due to the uncertainty and unknowns regarding their daily routines: “I never knew if I was going to my house or my grannies after school” (S8), “could never really plan much” (S7). Siblings also highlighted difficulties in living with a sibling with a CHC: “I was really annoyed at night, there was a whole night routine done with all the machines and everything was bleeping … we all got annoyed” (S7). This indicates the challenges in adjustment to new and daily routines whilst further indicating the emotional implications of change within the family. In addition, siblings reported struggling to adjust to their sibling’s condition: “I don’t know what it is, I would like a doctor to come and explain to me exactly what is wrong with her” (S6), “he can’t play games with me in case he is sore” (S5). For siblings who experienced physical relocation, other factors, such as changing schools and moving cities, were indicative of significant periods of adjustment: “I had to leave my friends behind” (S3), “I did have to move nursery schools” (S8), “We moved to a whole new area” (S8).

### 6.5. Communication

#### 6.5.1. Main Caregivers’ Perspective

Caregivers’ own doubts and uncertainties about adequately addressing their children’s emotional needs appeared to create barriers to open dialogue. There was a reluctance to broach sensitive topics articulated by caregivers and siblings. Caregivers reflected on the information the CHC child was provided in relation to their condition. Some caregivers reported, “he knew too much information … I think this scared him” (MC8), whilst others reported, “he didn’t understand what was happening, and I don’t think that helped” (MC8). This contrast is indicative of communication barriers between the CHC child, caregivers, and the medical team. Caregivers also reflected on the information provided to the child sibling, with the majority of caregivers feeling unsure if they had “done the right thing” (MC1,2,3,6,7,8) by communicating information to the sibling. The response from caregivers was dependent on sibling age, with siblings who were older in age posing more frequent questions to caregivers, which they felt should be answered: “as she got older, she asked more questions and always wondered where I was” (MC7). For younger siblings, caregivers report relaying “minimal information” due to their inability to comprehend and understand the situation: “I thought he was too young to understand” (MC5), “I wasn’t sure what she knew … but I’m sure she picked up on loads” (MC3). Caregivers report awareness of the barriers that prevented their children from discussing the impact of their experiences, as one caregiver articulated, “we now try and talk about things as much as possible as a family, but I am aware there are things they won’t tell me, and probably never will” (MC1). All families who participated in the study consistently reported they have now adopted open communication within the family, which has been noticed by siblings: “we realised we needed to talk about it more” (MC1,6,7,8).

#### 6.5.2. Extended Caregivers’ Perspective

Similarly to the main caregiver’s perspective, extended caregivers also grappled with doubts over the information communicated to their children. Some fathers reported, “I often think, did I spend enough time talking about it [with siblings] or was I avoiding it too” (EC5), “I thought the less she [CHC child] knew, the better” (EC3), “I underestimated how much [CHC child] knew about her illness and surgery, she picked it all up and it was quite shocking” (EC3). This data indicates the difficulties in communication that can occur driven by potential avoidance behaviours and uncertainties. Other extended caregivers expressed differing views: “they [sibling] were fine with it because it was all very well explained to them” (EC6,7,8), “I don’t think she [sibling] was impacted by not knowing, at least I hope not” (EC1). Acknowledgements were given to the difficulties children may have in processing the events of child hospitalisation: “I struggle as an adult, it must be incredibly difficult for a young child to process all of this” (EC6), “a challenge was [sibling] not really comprehending why she couldn’t see her sister” (EC3). Extended caregivers reflected on the importance of communication: “I think there needs to be more open communication between us and medical teams” (EC3), “for me, I would rather talk to a parent about it all rather than a professional” (EC2).

#### 6.5.3. Child Siblings’ Perspective

Communication also appeared to be a prominent theme of discussion amongst sibling participants. Some siblings reported, “normally I just talk to mum or I don’t really talk about it”, “It’s just a bit confusing because sometimes I don’t know what is going on“ (S6,7), “I think I got it more because I knew she was going to get an operation“ (S6). These reports highlight differences in family communicative strategies and the impact this has on siblings’ understanding. In contrast, a common thread amongst siblings apparent in the data was the reluctance to ask caregivers questions or to discuss their CHC sibling’s condition: “I don’t know whether to ask them questions or not” (S7), “mums explained it to me but not in much detail, because I don’t think she wants to explain it in a lot of detail” (S6), “I probably don’t know the whole of it, but I don’t want to ask” (S8). This suggests potential barriers to communication within the family and may be related to the emotional responses of caregivers about the topic.

### 6.6. ”Take-Home Messages” from Families

Main caregivers, extended caregivers, and child siblings have generously shared valuable insights into their experiences, offering perspectives on what has proven beneficial, what has not, and offering advice for fellow families facing similar circumstances, as well as services and policymakers within this area (Table 4).

## 7. Discussion and Limitations

This study demonstrates that the impacts of child hospitalisation extend far beyond the immediate medical context, creating layered effects. While the hospital stay itself is a defined event, its influence is systemic and enduring, shaped by the interaction of caregiver roles, gendered coping patterns, family resources, and the cultural norms embedded within healthcare environments.

Siblings were identified as particularly sensitive to shifts in the family’s emotional climate, often adapting their behaviours to ease caregiver stress. This attunement manifested in multiple ways, including emotional withdrawal, increased compliance, or intensified efforts to seek parental connection. Responses varied by age and developmental stage: older siblings were more likely to adopt quasi-caregiving roles, assisting with practical tasks or offering emotional reassurance to parents, while younger siblings more often expressed distress through behavioural changes or increased dependence. These patterns suggest that siblings are not passive observers but active participants in the family’s coping process, responding to the emotional transference generated by hospitalisation. Such variation emphasises the need for developmentally attuned interventions that recognise the differing capacities and coping strategies across age groups.

Grandparents played a distinct role in the family’s adaptation to hospitalisation, often balancing practical and emotional responsibilities simultaneously. Many provided direct support to parents, including caring for siblings, managing household tasks, or assisting with hospital visits while also processing their own emotional responses such as worry, sadness, and feelings of helplessness regarding the child’s condition and concern for their own child’s (parent) well-being. Their position within the family meant they were both contributors to, and recipients of, the emotional effects of hospitalisation. Thus, recognising grandparents as integral members of the caregiving network is essential, ensuring they receive appropriate information, emotional support, and resources to fulfil their role without becoming overburdened.

Gendered coping styles between main caregivers (mothers) and extended caregivers (fathers and grandfather) were also evident in the data. Mothers tended to express higher levels of overt emotional distress, whereas fathers more often adopted task-oriented coping strategies, prioritising stability through logistical and financial management. These patterns align with prior research [49,50] and likely reflect a combination of individual personality differences, cultural expectations, and norms reinforced within healthcare settings. Caregivers also differed in their preferred modes of receiving information: some sought detailed explanations to help reduce uncertainty, while others limited the information they received to avoid feeling overwhelmed. This is a crucial finding as without this awareness, one caregiver’s needs may be overlooked, potentially reducing engagement and increasing stress within the family system. If healthcare teams assume one uniform approach, they risk misinterpreting needs or overlooking concerns of the entire family system.

Variations in family experiences were also evident according to the nature of the child’s CHC. Families caring for children with medically complex or high-acuity conditions, particularly those requiring repeated surgical interventions or facing uncertain prognoses, described heightened ongoing vigilance, anticipatory grief, and intense emotions. These families reported greater disruption to daily routines and an increased need for frequent communication with their medical teams. In contrast, families of children with more stable conditions or predictable care pathways generally experienced lower levels of acute stress, though they still highlighted the cumulative mental and physical fatigue associated with repeated hospital admissions. The study also identified distinctions related to family resources and socioeconomic position. Families with greater financial stability, workplace flexibility, and social support networks were better able to cope with the logistical and emotional challenges of hospitalisation. They often had more autonomy in managing care schedules, could maintain a presence at the child’s bedside, and were able to access additional supports (e.g., paid childcare, private counselling). Families with fewer resources described increased difficulty in balancing work and caregiving, difficulty attending hospital appointments, and heightened stress related to housing or financial insecurity. These findings together emphasise the need for flexible, individualised communication strategies that account for developmental stage, type of CHC, socioeconomic realities, and cultural context. Consequently, standardised, “one-size-fits-all” approaches are insufficient.

Clinical applications based on these findings could include the implementation of family-centred rounds that actively invite questions from caregivers who prefer detailed engagement, alongside concise written summaries for those who favour less information. Additionally, initial assessments of each caregiver’s communication style, available resources, and cultural background should be supplemented by regular check-ins to adapt support as circumstances change. Practical assistance, such as financial counselling, flexible visitation policies, sibling care support, and culturally matched liaison staff, may be particularly beneficial for families facing socioeconomic or language barriers. Targeted interventions for siblings might include age-specific psychoeducation to normalise emotional responses, structured peer groups to reduce isolation, and liaison programmes with schools to address potential academic or social impacts. Grandparents, as dual-role caregivers, could benefit from dedicated support groups and information sessions that validate their experiences and equip them to assist without becoming overburdened.

### 7.1. Theoretical Framing 

The study’s findings align with systems theory [42], Bronfenbrenner’s ecological model [43], and Attachment theory [39]. Systems theory emphasises the importance of family dynamics and illustrates how hospitalisation disrupts family harmony, alters roles, and requires role reallocations. Bronfenbrenner’s model situates these processes within broader social and systemic contexts, including healthcare, education, and community support systems. For siblings, the educational impact was evident within the data, with numerous reports of siblings obtaining additional educational supports or receiving ASD/ ADHD assessments. This is a crucial finding that lends itself to research exploring the overlaps of traits between these diagnostics and experiencing traumatic or emotional responses [50,51,52,53]. This invites further investigation into the overlaps of trauma responses and developmental diagnoses [53,54,55,56]. Attachment theory provides an additional lens for understanding how secure relationships can buffer stress and may be disrupted by separation or shifting caregiving roles. When caregivers are overwhelmed by practical and emotional demands, their capacity for consistent, responsive care may diminish, with implications for attachment bonds. Theories of child cognitive development [4] and psychological frameworks such as CBT and ACT [56] complement this perspective by emphasising the role of cognition and meaning-making in adaptation and adjustment. Together, these frameworks highlight the need for multi-modal interventions addressing both emotional and cognitive processes within diverse family contexts.

### 7.2. Limitations and Future Directions

While the narratives gathered in this study provide rich insight into relational and systemic processes, the qualitative design inherently limits causal interpretation. Although hospitalisation was frequently associated with relational difficulties, it remains unclear whether hospitalisation directly precipitates marital breakdown or merely amplifies pre-existing difficulties. Future studies would benefit from longitudinal designs, tracking families before, during, and after hospitalisation to explore causal pathways, and examining how factors such as marital relationships, sibling age, type and complexity of the child’s CHC, family resources, and cultural background shape outcomes.

The findings are based on reflections from eight families sharing their experiences, cautioning against external validity and applicability within other cultures and healthcare systems. The findings therefore are only indicative for situations and families having similar characteristics. In attempts to balance this, the recruitment purposefully did not focus on a homogeneous sample. Participants varied in age, gender, and crucial elements of the CHC child’s diagnosis, prognosis, and surgeries. It is possible that those who participated were more willing to share and better adjusted to their experiences and child’s condition, potentially influencing findings. Despite these limitations, common themes emerged across narratives, offering invaluable insights.

Conducting interviews with child siblings warrants further considerations for future research due to varying developmental and communicative abilities. The presence of a parent during an interview with a child under 16 can provide comfort and safety for the interview process; however, it may also influence the child’s responses. This can pose challenges to data reliability and ethical considerations due to balancing the need for parental consent with the child’s right to privacy and autonomy.

Chronological age influenced the direction of inclusion criteria, with little consideration given to developmental ages. As previous research posits, the effects of trauma can impact neurological and relational developments [57], therefore, the siblings of hospitalised children may experience continuous emotional difficulties or may not have the ability to describe their experiences, complicating the interview process. In addition, qualitative research is susceptible to researcher subjectivity and small sample sizes, which can influence the interpretation, credibility, and validity of the findings. Although steps were taken to minimise bias, researcher LM being a trainee clinical psychologist may have shaped the nature of follow-up questions and participant responses, leading to socially desirable answers. While this study was not designed to examine cultural or socioeconomic differences, the sample lacked broad diversity in these domains, limiting the generalisability of findings. Nonetheless, some variation was observed in how families coped with practical challenges: self-employed caregivers often described greater flexibility in managing hospital visits and caregiving responsibilities, whereas those employed in more structured roles sometimes faced additional stress due to limited employer accommodation. These observations highlight the need for future research to explore these areas more comprehensively.

While some areas for future research have been identified, the theoretical frameworks guiding clinical interventions and evidence-based practices are already firmly established. Therefore, these findings serve as additional supportive evidence, warranting immediate implementation within clinical paediatric practices. This involves advocacy for securing funding, widening and expanding access to services, adopting community-focused models, enhancing support systems for siblings and families, and innovating systemic and holistic approaches. The key “take-home messages” and advice shared by participants is the most influential and applicable aspect of the research. Listening to and sharing these messages within services offers compelling suggestions for change, calling for a restructure of existing frameworks which focus more on implementing intervention and prevention models within paediatric healthcare and to the family system as a whole.

## 8. Conclusions

Families’ lived experiences are essential to shaping the future of healthcare. Listening to and acting on their feedback reveals gaps, inequities, and communication barriers. Ultimately, the message is clear: families, particularly siblings, need better holistic support services, understanding, funding, and practical supports worldwide to navigate the complexities of child hospitalisation. Urgent structural, cultural, societal, and systemic changes are needed to provide holistic, equitable support. It is time to transform paediatric healthcare by listening to families, closing gaps, and ensuring comprehensive, child-centred support.

## Figures and Tables

**Table 1:** Participant characteristics.

Participant Group	n	Description
Primary caregivers	8	All identified as mothers
Extended caregivers	7	Fathers and grandparents
Child siblings	8	Aged 4–16 years
Total	23	

**Table 2:** Inclusion and exclusion criteria.

Inclusion Criteria	Exclusion Criteria
1. Family has a child who experienced recurring hospitalisation with a chronic medical illness. 2. Child is not currently in hospital. 3. Family and child are residents in Northern Ireland. 4. Hospitalised child must have at least one sibling between the ages of 4 and 18. (The minimum age requirement to participate in the study will be age 4 and above. This is due to the necessity for participants to be able to engage with the interview process and communicate effectively.) 5. More than one caregiver involved in caring for the sibling(s). 6. Child sibling who is able to communicate and willing to take part in the interview process. 7. English speaking.	1. Families who currently have a child in hospital. 2. * Families or persons with a significant mental health history directly related to the hospitalisation process. This is to prevent further distress and guided by ethical considerations. 3. * Families or persons currently receiving active support for their mental health related to child hospitalisation. 4. Families experiencing end-of-life care. This is to prevent further distress and guided by ethical considerations. 5. Families or persons who do not wish to participate. Families and individuals who do not wish to or are deemed unsuitable to participate in the study will be provided with a list of resources to contact for support. 6. Families who reside outside of Northern Ireland.

* Participants who required support for their mental health directly relating to the hospitalisation experience were excluded from the study. This involved asking participants if they had or do currently attend adult mental health services or related services for support with the child hospitalisation. This decision was guided by QUB Ethics Committee to prevent participant distress.

**Table 3:** Summary of participant groups and key themes.

Participant Group	Number (n)	Key Themes Identified
Primary caregivers	8	[Emotional impact], [Relational impact], [Practicalities], [Adjustment for family system], [communication], [Take-home messages]
Extended caregivers	7	[Emotional impact], [Relational impact], [Practicalities], [Adjustment for family system], [communication], [Take-home messages]
Child siblings	8	[Emotional impact], [Relational impact], [Adjustment for family system], [Communication], [Take-home messages]

**Table 4:** Advice for families and professionals.

Caregivers’ advice for families	It’s crucial to hold onto hope… it’s astonishing what our children can endure and overcome”. Remember, it’s okay not to bottle up your emotions … don’t hold it all in. As hard as it is, prioritise your sleep. If you don’t have sleep, you have nothing left to give. Simple acts like batch cooking ease the stress. Building a strong support network is important… don’t hesitate to reach out for help. Although it can be hard sometimes, keeping communication lines open with the medical team is good… you get strength knowing that they’re battling for your child too, you know, there’s good people in there. Lean on each other, and don’t be afraid to ask for the support you need. Make connections with other people through charities… you could be friends for life. A doctor once said to me, when [CHC child] recovers, what life have you got? So think of the bigger picture… concentrate on where you want to get to and not just on where you want to be at the end of the storm… keep your lives running.
Caregivers’ advice for services	Sometimes there is too much information … I can’t tolerate that. I won’t remember what you’ve told me. Have consistent people/ medical teams for the children who are going to be in hospital longer… it is really needed. Having access to facilities for parents on the wards would be great… somewhere to go and sit for a minute or to make a cup of tea. Offer mindfulness classes through services, either in the trust or outside … I think this helped me the most. More groups to connect with other parents and for our other children. In hindsight, help with managing anxieties about the whole hospital experience after discharge … I know at the time [during hospitalisation] I would of struggled with this. I think there needs to be more done to help a person navigate the system when they go in … if there was some kind of brochure that told them who everyone is, what time the shift will change, where is the canteen and its opening hours … Tell them that, it would help. I want staff to know, we don’t mean to be hard work, but we are suffering in our own way. I think the focus is obviously on the sick child, but nobody asks about the other children in the house … I would like someone to ask if they are ok too. A leaflet or anything with some key phone numbers on it, some people who can provide information about benefits, about what you are entitled to in times of need. Financial support to help with hidden costs of food, vending machines, car parking tickets, clothes, petrol, oil heating at home. A workshop like a health and wellness day for parents and carers that could give people some tools to deal with events as they happen … Like having a toolbox of strategies that might work for me. Sometimes I would prefer to talk to parent rather than a professional.
Siblings’ advice for other siblings	Don’t be worried, just think that it’s gonna be OK. Try to have Positive thoughts. No negative thoughts. Just always be close to them. It’s hard for everyone, even your sibling… remember that.

## Data Availability

The data presented in this study are not publicly available due to ethical restrictions and to protect participant privacy. Data may be made available on reasonable request from the corresponding author and subject to approval by the QUB ethics committee.

## Supplementary Materials

The following supporting information can be downloaded at: https://www.mdpi.com/article/10.3390/children12091159/s1, Supplementary Material SA: Children’s Health Coalition permission form; Supplementary Material SB: Study Advertisement; Supplementary Material SC: Participant Information Sheet; Supplementary Material SD: QUB Ethical Approval; Supplementary Material SE: Distress protocol; Supplementary Material SF: Debrief form; Supplementary Material SG: Interview Guide & Visual Supports protocol; Supplementary Material SH: Framework matrix; Supplementary Material SI: Eligibility criteria; Supplementary Material SJ: QUB Policies; Supplementary Material SK: Consent forms.
