# Supplementary material for: Exploring the Impact of Child Hospitalisation on the Family System: A Qualitative Study Using Framework Analysis
Source: Children (Basel). 2025 Aug 31;12(9):1159. doi: 10.3390/children12091159 (PMC12468557; doi:10.3390/children12091159)
Supplement: Supplementary file 1 [file children-12-01159-s001.zip › children-3760051-supplementary.pdf]

Technical Supplementary Material

Supplementary Material SA: Children's Health Coalition permission form

Supplementary Material SB: Study Advertisement

Supplementary Material SC: Participant Information Sheet

Supplementary Material SD: QUB Ethical Approval

Supplementary Material SE: Distress protocol

Supplementary Material SF: Debrief form

Supplementary Material SG: Interview Guide & Visual Supports protocol

Supplementary Material SH: Framework matrix

Supplementary Material SI: Eligibility criteria

Supplementary Material SJ: QUB Policies

Supplementary Material SK: Consent forms

Supplementary Material SA: Children's Health Coalition signed recruitment statement

QUEEN'S UNIVERSITY BELFAST

NI Children's Health Coalition

<u>Children's Health Coalition – Recruitment Involvement</u>

Statement of permission

We at the Northern Ireland Children's Health Coalition will support and aid in the recruitment of participants for the research project 'Exploring the impact of Child Hospitalisation on the Family System', conducted by Lauren Murray, Trainee Clinical Psychologist at Queens University Belfast and her supervisors, Professor Pauline Adair, Dr David McCormack and Professor Nicola Doherty. We agree to disseminate and advertise this project through our organisations social platforms and agree to be a named organisation on the project's debrief form. This means that participants who wish to seek support post study can contact the Children's Health Coalition organisations for further information and/or support.

Date: 27/01/2023

Print Name: Alison McNulty & Joanne McCallister, Co-Chairs of Children's Health Coalition

Signature:

Supplementary Material SB: Study Advertisement

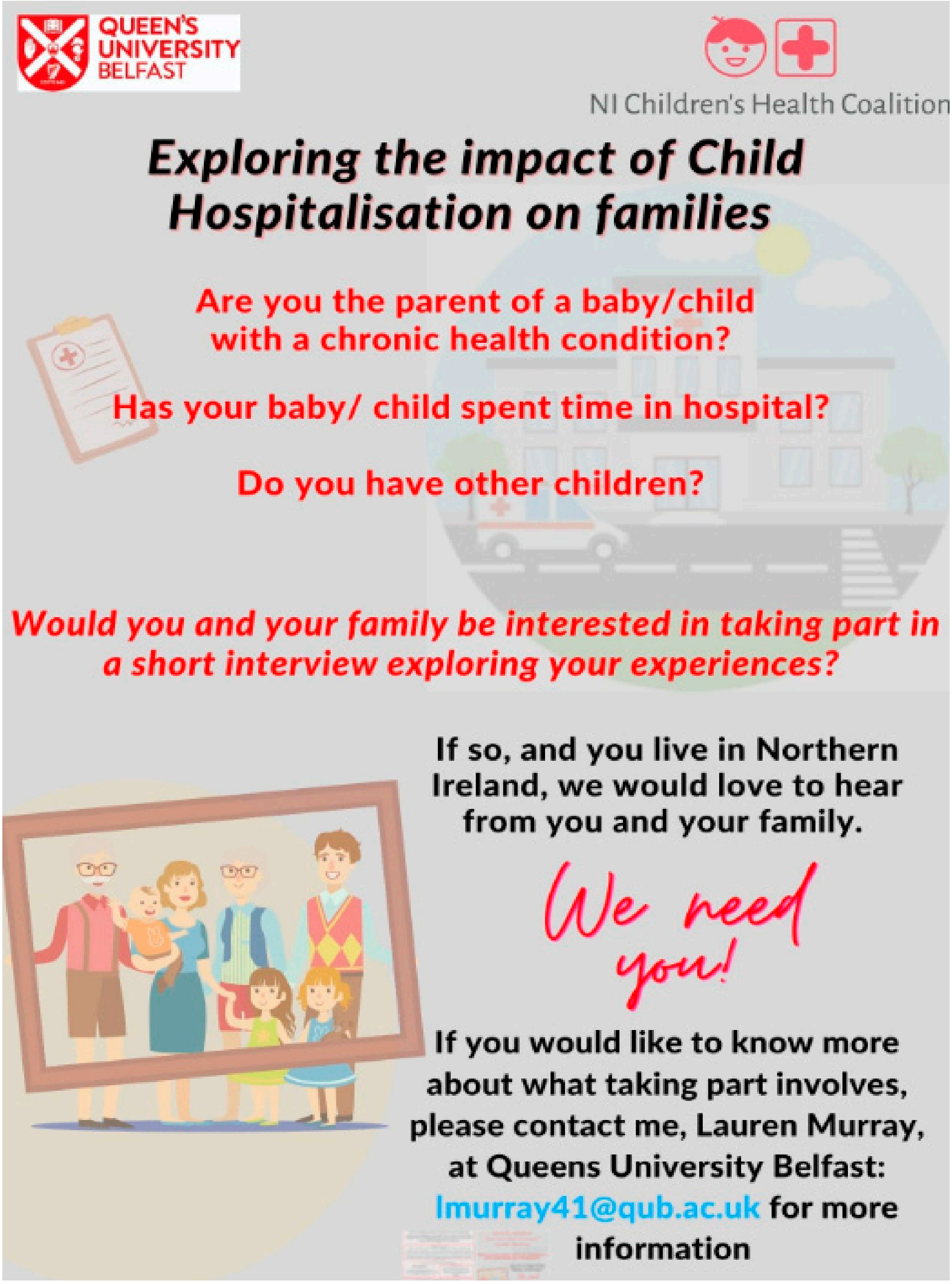

Supplementary Material SC: Participant Information Sheet

QUEEN'S UNIVERSITY BELFAST

NI Children's Health Coalition

Appendix A – Information leaflet

Information Leaflet

Thank you for expressing your interest in this research:
Exploring the Impact of Child Hospitalisation on the Family System

Purpose of the research

Having a child in hospital and can cause a lot of disruptions to family routines and life in general. When there are prolonged or multiple hospitalisations, such as for children with chronic health conditions, this impact can be greater. We need to understand how best to support families by talking to them directly about what they need as well as understanding the real impact of a child being in hospital on family life. This involves not just talking to parents/caregivers but others who may be impacted such as siblings and other caregivers who help during hospitalisation.

What will I need to do?

This study will involve taking part in an interview to talk about your experiences of having a child in hospital. The study will require an interview with a parent/ caregiver and other members of the family involved in caring for other children you may have whilst your child is an inpatient. This research study also hopes to have an informal discussion with siblings of the child in hospital to provide them with a safe and open space to share their experiences. The parent/ caregiver will be present with the child during this interview if they are under the age of 16. Children aged 16 years and above have the option for an adult to be present.

You can choose from the below interview processes…

Face to face interview

This interview will be conducted in person, at Queens University Belfast. Another suitable location can also be discussed with the researcher if preferred.

OR

Online interview

This interview will be conducted using Microsoft Teams video platform. This will follow a similar structure to the face-to-face interview, only can be completed from your own home at a time agreed with the researcher

All interviews for participants aged 16 years and above will last no longer than 60 minutes. Interviews for children below the age of 16 years will last no longer than 30 minutes.

You will now be contacted by the researcher via telephone to discuss further and arrange an interview time that suits you and your family. Should you wish to request further details regarding this study, please do not hesitate to contact me via email on the below details; ***Lmurray41@qub.ac.uk***

Your agreement to be contacted or request more information about the study does not obligate you to participate in any study. If you would like any additional information about this study, please contact members of the research team on Lauren Murray – lmurray41@qub.ac.uk. **This research will be conducted in compliance with data protection legislation. For more information about how we look after your information, how to access your rights and who to contact if you have any queries or concerns about data protection please visit the Queens University Belfast website on: https://www.qub.ac.uk/privacynotice/Research/ListofResearchPrivacyNotices/PrivacyNoticeforResearchParticipants.html**

Supplementary Material SD: QUB ethical approval

QUEEN'S UNIVERSITY BELFAST

**Date:**	21 April 2023
**To:**	Professor Pauline Adair
**Faculty REC Reference Number:**	EPS 23_17
**Full Title:**	Exploring the impact of child hospitalisation on the family system
**Decision:**	**APPROVED**

Thank you for your response to Committee review of your application which was reviewed at the meeting of the Faculty of Engineering and Physical Sciences Research Ethics Committee (EPS Faculty REC) on 08 February 2023.

Your response and revised supporting documents were considered and some clarification and revisions were requested on 15 February 2023 and 27 March 2023. You submitted the requested information on 05 April 2023 and this was forwarded for review.

The response has been reviewed and deemed satisfactory. The application has been **approved**.

Conditions of the Approval

The Faculty REC approval is subject to the following conditions:

(i) The study must be conducted in accordance with all relevant legislation. All relevant management approvals from organisations involved in the research must be obtained.
(ii) When the research involves human volunteers the study must be entered on the University's Insurance Database.
(iii) Monitoring and auditing process must be complied with including submission of annual progress reports to the Faculty REC.
(iv) Any face to face study activity is subject to the submission, approval and adherence to a COVID-19 Risk Assessment.

It is the Chief Investigator's responsibility to ensure the study is conducted in accordance with the conditions stipulated.

Any future changes to any part of the submitted application, protocol or supporting documentation must be notified to the Committee prior to these changes taking place.

Approved Documents

The documents approved by the Faculty REC are listed in the table below.

Documentation Received	Version	Date
Application Form (Revised)		Received 05 April 2023
Cover Letter Addressing Committee Comments		Received 15 March 2023
Cover Letter Addressing Committee Comments		Received 05 April 2023
Research Protocol (inc Appendices)	2	13 March 2023
Appendix A Participant Information Sheet	2	13 March 2023
Appendix B Interview Schedules		Received 05 April 2023
Appendix C Visual Aids for Interview		Received 05 April 2023

Appendix D Inclusion and Exclusion Criteria	2	13 March 2023
Appendix E Distress Protocol	2	13 March 2023
Appendix F QUB Policies	2	13 March 2023
Appendix G Consent Form and Assent Form	2	13 March 2023
Appendix H Debrief	2	13 March 2023
Appendix I Study Advertisement	2	13 March 2023
Appendix K Peer Review	2	13 March 2023
Appendix L Proposed Project Timetable	2	13 March 2023
Appendix M Children's Health Coalition Permission Form	2	13 March 2023

If you would like to discuss this further please contact the Research Ethics Officer, Mr Stefan Curran, at facultyreceps@qub.ac.uk.

Yours sincerely

pp Professor Brendan Murtagh
Chair, EPS Faculty REC

Supplementary Material SE: Distress protocol

The protocol for managing distress in the context of a research interview

(Modified from: *Draucker C B, Martsolf D S and Poole C (2009) Developing Distress Protocols for research on Sensitive Topics. Archives of Psychiatric Nursing 23 (5) pp 343-350)*

Distress

- A participant indicates they are experiencing emotional distress or exhibits indicative behaviours such as crying, shaking etc.

Response

- Stop the discussion/ interview
- Check in with the participants and offer support through using a listening ear and providing containment.
- Assess participant welfare.
- Remind participant of their right to withdraw up to 4 weeks post study, and cease interview if requested. If participant wishes to continue, interview can resume.
- Provide participant with debrief form containing a list of numbers to contact and encourage discussion with GP or mental health provider.
- Discuss with research team, containing qualified Clinical Psychologists and follow advice.
- Offer participant a follow up call if deemed appropriate to do so and the participant consents to this.

Supplementary Material SF: Debrief form

QUEEN'S UNIVERSITY BELFAST

NI Children's Health Coalition

Debrief

Thank you for taking the time to participate in this study and sharing your experiences. The primary aim of this research was to explore the overall impact of child hospitalisation on the family system and allow your experiences to be heard. The information you have provided will be extremely useful in helping us to develop new ways of supporting children and families in similar circumstances.

All information you have provided will be kept anonymous and confidential. The researchers will identify themes from the interviews to support the study, thus specific information gathered during the interview stage will be unidentifiable. You have the right to withdraw all information you provided to the study up to 4 weeks after participation. Should you wish to withdraw, your details and interview data will be permanently erased.

We understand that talking about difficult experiences can be upsetting and cause you and your family distress. Should you wish to seek support after this study, please contact the below services;

- Parenting NI on **0808 8010 722**
- Childline on **0800 1111**
- Samaritans' helpline on **116 123** (free of charge)
- Lifeline (Northern Ireland) on **0808 808 8000**
- Children's Health Coalition organisations (Please see overleaf).
- Research team member, Professor Pauline Adair (Chief investigator; Clinical Psychologist) on **028 9097 4353**

The results of this study will be disseminated to the Children's Health Coalition and submitted for publication to relevant journals. Participants will have access to a lay summary of the results when the study is finished. This will be made available through the Children's Health Coalition for any participant who wishes to access this. It is hopeful that the research will be published and able to be accessed by the public. If you have any queries or want to find out about the final results of this study, you should contact the researchers via email; lmurray41@qub.ac.uk
Many thanks for your participation and best wishes for the future,

Lauren Murray.
Trainee Clinical Psychologist, Queens University Belfast

Children's Health Coalition Organisations

The Children's Health Coalition contains various organisations that can offer you and your family support in different areas. Please contact the organisation which you feel could support you/ your family best.

TinyLife

Supporting Families of premature and sick babies who start life in a neonatal unit.
Telephone: **02890815050.** Email: info@tinylife.org.uk

Children's Heartbeat Trust

Supporting children with congenital heart disease and their families.
Telephone: **028 9031 2228** Email: info@childrensheartbeattrust.org

Child Brain Injury Trust

Supporting children, young people and families affected by childhood acquired brain injury.
Telephone: **02890817145**

Mae Murray Foundation

Supporting people of all ages and abilities to take part in social and leisure settings.
Email: info@maemurrayfoundation.org/03006001166

Contact

Contact, the charity for families with disabled children. We understand that life with a disabled child brings unique challenges, and we exist to help families feel valued, supported, confident and informed.
Email: nireland.office@contact.org.uk
Helpline: **0808 808 3555** (Free of charge)

Cancer Fund for Children

Supporting children and young people impacted by cancer.
Telephone: **028 9080 5599**
Email: services@cancerfundforchildren.com

Shine Charity

Supporting members, their families and professionals with the conditions of Spina Bifida and /or Hydrocephalus.
Telephone: **07789 616420** or email firstcontact@shinecharity.org.uk

The Children's Cancer Unit Charity

Supporting the Children's Haematology & Cancer Unit at the Royal Belfast Hospital for Sick Children.
Telephone: **077 1881 8134**

Family Fund

Provide grants to families raising disabled or seriously ill children and young people.
Telephone **07818 456378** or contact Emma on emma.mckeown@familyfund.org.uk

Brain Injury Matters

Supporting all those impacted by acquired brain injury including children, young people and their families.
Telephone 028 9070 5125.
Email: info@braininjurymatters.org.uk

Angel Eyes NI

Supporting Children with Vision Impairment, and their families, across Northern Ireland.
Email: info@angeleyesni.org
Telephone: **07502265152**

Supplementary Material SG: Interview guide and visual supports protocol

Interview 1 – Parents/ Main Caregiver

Relationship to child: …………………………………………………………………….

1. What is it like for you and your family when (Name of child) is admitted to hospital?

2. What impact, if any, does a planned admission have on you and your family?

Prompts: explore practical, emotional and financial implications

3. **What impact, if any, does a sudden admission have on you and your family?**

Prompts: explore practical, emotional and financial implications

4. What are the benefits, if any, of your child being hospitalised for you and your family?

5. What are the challenges, if any, of your child being hospitalised for you and your family?

Prompts: explore practical, emotional and financial implications

6. Is there any financial implications or hidden costs for you and your family when (name of child) is admitted to hospital?

Prompts: Petrol, carparking, reduced time in work etc. Anything you had to give up due to finances? What is the emotional impact of this?

5. Can you tell me how (name of child) being in hospital affects other family members?

Prompts: Was there anything you noticed? Anything you felt was helpful/ unhelpful? Is there anything that professionals could of done with (name of siblings) to help?

6**. What does it mean for you and your family's relationships to have a child who requires Hospitalisation?**

Prompt: Anything you noticed between relationships/ family dynamic.

7. What do you think would benefit other families going through similar experiences based on your own experiences?

Prompt: If you had to choose one piece of advice for families going through similar experiences as you, what would that be?

<u>Interview 2 – Child Siblings (ages 4-18)</u>

Relationship to child: ……………………………………………………………………

All questions will be adapted based on age and visual prompts used as necessary

1. **What is it like when (name of child) has to go to hospital?**

   Prompts: What was home life/ school life/ social life like at this time?
   What was it like for you being away from X and family?

2. ***The next question is about your feelings -*** **How do you feel about it?**

   Prompts: Visual aids (feelings poster, Mr.Fox poster) What words/ colours jump to mind for you? Do you feel anything in your body?

3. **Was there anything you liked about it/ found helpful?**

   Prompt: What do you not like about it/ found unhelpful?

4. **Do you know when (name of child) is going into hospital? – I wonder what that is like for you?**

   Prompt: What is it like for you when (name of child) has to go into hospital suddenly?

5. **What was helpful/ unhelpful when (name of child) was in hospital?**

   Prompts: was there a family member that became really important to you? Did you get enough information from mum/ dad/ others/ professionals? Did your friends notice a difference in you when (name of child) was in hospital? Did you ever miss out on anything?

6. **What do you think would help you/ other boys and girls that have a brother/ sister that is sick and has to go to hospital?**

Prompt: Is there anything you would like to say to other boys and girls?

Did anyone ever ask how you felt about everything?

Adaptive language for 'Feelings Poster'

1. When (name of child) has to go to hospital – how do you feel? How did you feel being away from family? What do you feel most of the time when X is in hospital? (Point to poster)
2. If you had to pick a face to tell me how you feel about X in hospital, which face would you pick?
3. When X had to go into hospital and you didn't know – how did you feel? What face would you choose to tell me how you felt?
4. Was there anything you liked about it? (point to happy face). Anything you didn't like about it? (point to sad face). How did other family members make you feel when X was in hospital?

Adaptive Language for 'Feelings of Mr. Fox

1. Oh my, Mr. Fox's (brother/ sister) had to go into hospital- how do you think Mr. Fox is feeling? Point to poster
2. Mr. Fox didn't know his (brother/ sister) was going to hospital! How do you think he was feeling when he found out?
3. Mr. Fox's (brother/sister) had to go into hospital before. Mr. Fox knew about this. How do you think Mr. Fox felt when he knew about his brother having to go to hospital?
4. Do you think was there anything that Mr. Fox liked about his brother/sister being in hospital?
5. Do you think was there anything Mr. Fox didn't like about his brother/ sister being in hospital?
6. Mr. Fox really wants to help other foxes if they ever have a (brother/ sister) that has to go to hospital. What do you think Mr. Fox could tell them?

<u>Interview 3 – Other caregiving family member (largely involved family member)</u>

Relationship to child: ……………………………………………………………………

1. **Can you tell me how (name of child) being in hospital affects other family members?**

   Prompt: What did you notice? Challenges/ benefits for the family?

2. **The next question is about you: What was this experience like for you?**

   Prompt: How did you cope?

3. **Are there challenges/ benefits for you when (name of child) is hospitalised? If so, what are they, what is the impact of these?** *eg. Protecting others/ strong/ time demands/ feeling split/ missing family.*

   Prompt: does this differ if the admission is planned or sudden?

4. **What advice would you give other families going through similar experiences?**

   Prompt: Is there anything you would of liked to have known? What would of made things easier for you?

<u>Protocol for using visual supports in interview</u>

Visual supports will be used to aid the interview process with children under the age of 16 throughout this study. Visual supports aim to support communication with children and provide an alternative mode of communicating with the researcher.

A recent study conducted by Siying et al., (2021) on infant development found that children as young as 30months were able to recognise and understand the emotional value of emotion symbols using virtual images. This study adds to copious research concluding that this ability emerges in early development, unless development has been disrupted by extraneous circumstances.

Adhering to literature on child development, two types of visual supports will be available for use within this study.

1. The ‘feelings faces’ poster (see below) will be used for all children participating in the study, from ages 4-15 years. Only if the child sibling appears to struggle to answer the questions outlined in the interview schedule, will this visual aid be used as a prompt. The researcher will then ask the same question again, whilst presenting the child with the ‘feelings faces’ poster. The researcher will point to each face and read

out the description of the face below (See poster). The researcher will ask the same question again whilst using the prompt to help the child answer, either by verbally responding or pointing to a face in the 'feelings poster'.

2. The second visual aid poster is 'Feelings of Fox'. This will only be used if the child sibling displays difficulty answering the questions outlined in the interview schedule whilst also using the "feelings faces' visual aid. The researcher will point to the faces of the fox and read out the description of the face below (see poster). The researcher will then ask the same question with the use of adaptive language. This means, instead of the researcher asking individualised questions to the child sibling, the questions will be interpreted through the use of 'Mr. Fox' and adapted through use of therapeutic language. This technique is well established throughout Clinical Psychology Research and practice when working with children. For example, instead of asking the child, '*what is it like when (Name of child) has to go into hospital?'. The researcher will ask 'Oh my, Mr Fox was told his brother had to go to hospital. What do you think Mr Fox is feeling?'. The researcher will point to the 'feelings of fox poster' as a prompt when asking this question.*

3. The interview schedule for the child siblings has been amended to reflect this adaptive language.

FEELINGS FACES

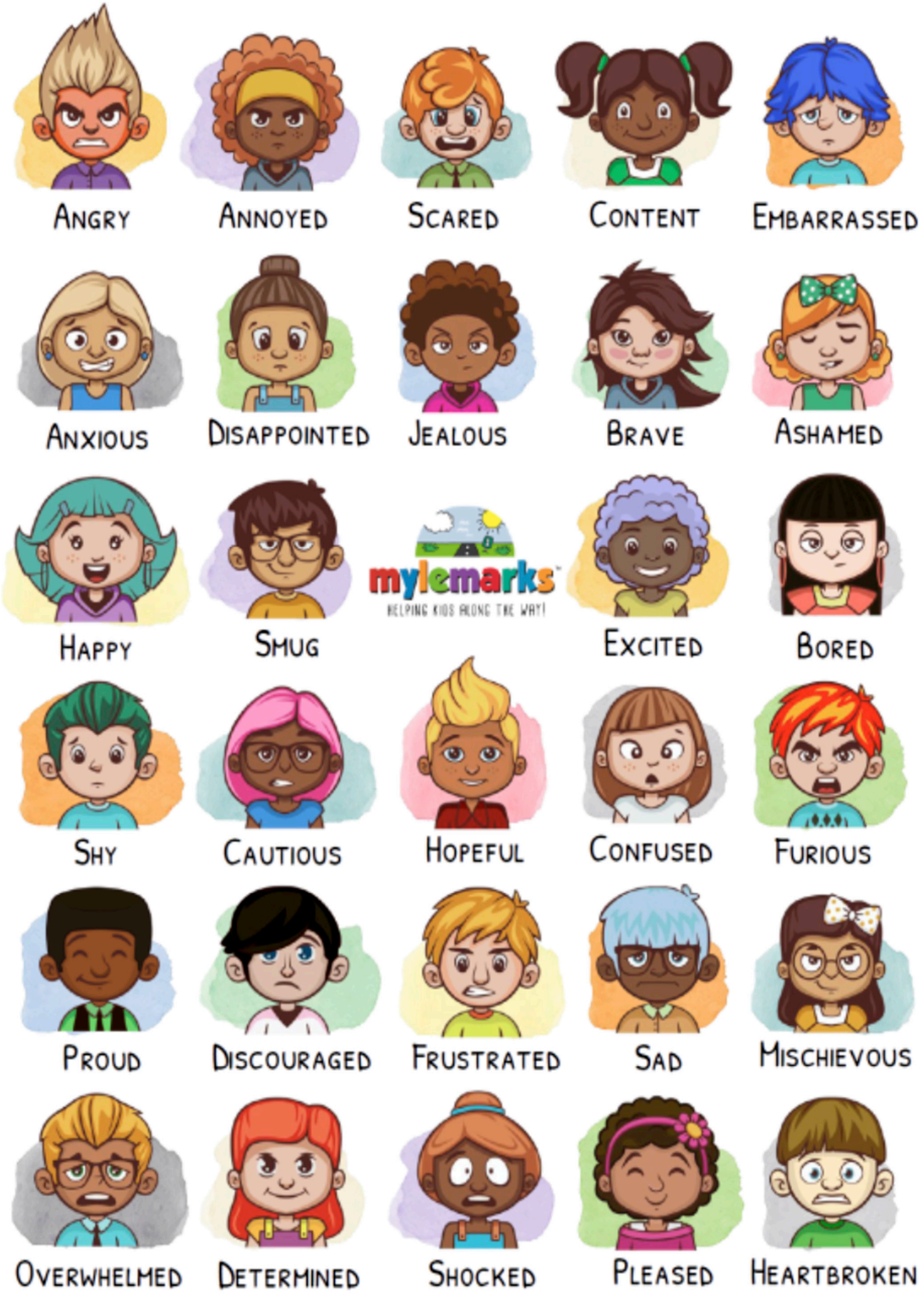

© 2020 Mylemarks LLC. All Rights Reserved.
For more resources, visit www.mylemarks.com! Watercolor Graphic by Sarah Pecorino Illustration:
https://www.teacherspayteachers.com/Store/Sarah-Pecorino-Illustration

Feelings of Fox

How are you feeling today?

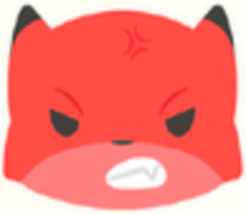

ANGRY

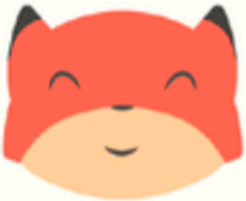

CALM

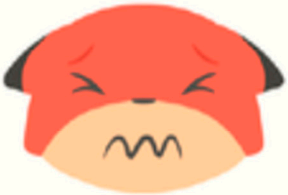

FRUSTRATED

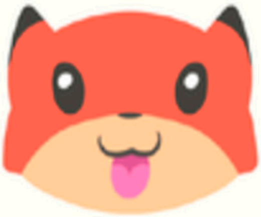

HAPPY

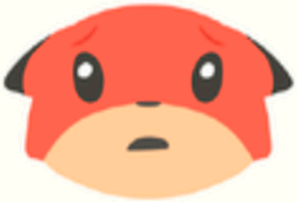

NERVOUS

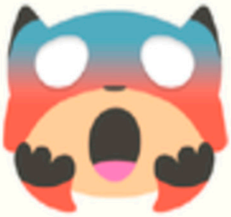

SCARED

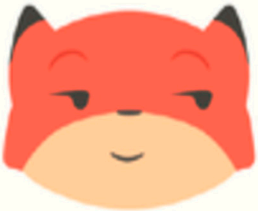

SHY

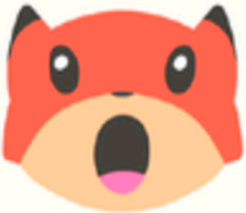

SURPRISED

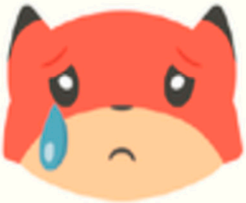

SAD

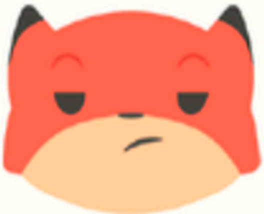

BORED

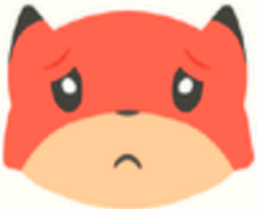

LONELY

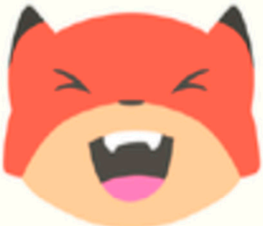

EXCITED

mylemarks

© 2022 Mylemarks LLC. All Rights Reserved.
For more resources, visit www.mylemarks.com!

Supplementary Material SH: Example of the ‘Framework Matrix’

Framework Matrix Impact of child hospitalisation on the family system

**Matrix codes**	
Main Caregiver	MC
Extended Caregiver	EC
Child Sibling	S
Family references (Anonymised)	Numbers 1 – 8
Redacted information	[redacted]

**Colour code**	**Codes**	**Overarching Theme**
	Traumatic impact of repeated hospitalisations	Emotional Impact
	The Unknowns	
	Coping skills/ styles	
	Ongoing impacts	
	Difficulties in adjustment	Adjustment for the family system
	The new normal	
	Parents as medical experts	
	Bonding in the hospital setting	Relational Impact
	Relational maintainers	
	Relational disrupters	
	Finance and Employment implications	Practicalities
	Environmental implications	
	Service implications	
	Practical benefits	
	Advice for other families	‘Take home messages’
	Advice for staff/ professionals	
	Advice for other child siblings	
	Communication styles	Communication
	Communication barriers	

Preliminary Codes	Extract	Notes and ideas	CODE and description
Multiple traumatic experiences	He had 29 hospital stays. And then I stopped counting cause I just couldn't count anymore. So I actually don't know how many he had. Maybe close to 40, but I stopped at 29 and like they were all quite traumatic. – *MC1*<br><br>She was eleven months old before she even came home from hospital. So we were in and out three, four times on a good year. *MC2*<br><br>There was an end-of-life care when she was a small baby. So I go back there quite a bit, you know, when I get very anxious, I go to that point again and go, are we going back to that again? *MC2*<br><br>I definitely never have been diagnosed because I never went and seen about it, but I definitely would have PTSD from her early experiences. *MC4*<br><br>I didn't expect to be faced with a baby with a diagnosis or faced with the, you know, NICU life for five weeks. *MC1*<br><br>Just trying to stay with my baby in hospital in NICU was horrendous. Still, absolutely hate thinking about that time. *MC1*<br><br>I'll feel sick for days – knots in tummy. Take about a week post hospital to come back to normal- jittery and all that kind of feeling. *MC1*<br><br>Once you know the next one's coming, I can just feel that kind of anxiety start to rise again. *MC6*<br><br>Can't watch TV shows like greys anatomy – triggering. *MC7*<br><br>I have to watch videos of the surgeries – I need to know what's happening. I needed to see what they were doing. I just need to be aware. *MC6*<br><br>What's going on in my head is 10 times worse than what's happening in that room. *MC6*	Became the new normal	Traumatic impact of repeated hospitalisations<br>Perceived experiences of traumatic events including witnessing medical procedures, repeated hospitalisations, separation from baby.<br><br>Emotional impact

Preliminary Codes	Extract	Notes and ideas	CODE and description
(horrific) period after birth – ICU, separation from baby, rollercoaster of emotions from hope to horrific. Witnessing medical interventions.	There was problems and nobody believed me to be frank initially – whenever I found out she had [redacted] I felt a wee bit of a relief from my shoulders, to know that actually, it wasn't in my head and this was the cause. *MC4*<br><br>Chaotic all the time as a family. *MC1*<br><br>Just wanted to survive and make sure everyone else around you was surviving. *MC1*<br><br>For me, the impact depends which hospital I am going to. In one hospital, they are maybe more open to what I feel as a parent. And this helps. *MC2*<br><br>I've definitely had depression with fear and worry, really irritable or feeling really fatigued. I could just want to go to sleep all the time, you know? *MC1*<br><br>I feel sick in the queue for the car park. *MC6*<br><br>it's just panic, you know, it was just complete anxiety. My throat was closed and I couldn't breathe. I thought my heart was palpitating, you know, physiologically at all the psychological symptoms. So I think it really was very hard on my body, you know, because mentally you have to hold everybody else up. *MC4*<br><br>I think you do get PTSD, but you don't get it straight away. So like, after we come home from that double surgery, I started getting pain and you know, like kind of the gallbladder area and then way up high in the tummy and it was getting checked through and they were like no, you're fine, you're fine. But again it must have been like a nervous reaction – *MC1*<br><br>So as soon as he was born, he was transferred hospitals and we were transferred three times, actually, throughout his neonatal stay. And that was 38 days. So after 38 days, he was allowed home. But it was probably our most traumatic as you could maybe imagine, you know, brand new baby. You know, it wasn't ideal that he was being moved around so much. *MC1*		Relational impact

Preliminary Codes	Extract	Notes and ideas	CODE and description
Trained in giving medications and machinery and resuscitation - not a thing to do to a brand new mummy<br><br>Understanding traumatic impact on child with CHC	When he was born, he was fine for like 50 minutes. And we were like, Holy God, this is great. He seems great. And then he just flat lined and had to be resuscitated and the full works. So that day, after the 50 minutes, we didn't see him again until that night, cause they worked on him the whole day. And they brought me up to the maternity ward with all the babies and mummies. And it was just horrific.<br>So I was literally sitting in the hospital by myself. Nobody was with me. - *MC1*<br><br>And the last thing they did was at like 11:00 o'clock at night before we took him home, was being trained to resuscitate him. And I was just like, this is not a thing to do to a brand new mummy like – *MC1*<br><br>It was awful because they had asked twice, did we want him christened? And to me, this seemed like, you know, a major threat, cause they weren't just like, do you want to get him christened? They were definitely telling me that, you know, he was probably going, we were gonna lose him- *MC1*<br><br>And he really has a lot of like, basically post-traumatic stress, you know? he would never be a settled boy in hospital. You know he wouldn't eat. He would barely sleep, you know, he was very nervous of people coming around. - *MC1*<br><br>Like I think that like [redacted] a bit like me but I would have a lot of like post traumatic stress around the hospitals now too. And I would nearly do anything to avoid it. You know, I really would. It just brings out the worst in us all. - *MC1*<br><br>When things slowed down, it really hit us and we're probably still processing those difficult few years because both hospital admissions and outpatient appointments he still has a lot of- *MC1*		Ongoing impact<br><br>Coping skills// styles

Supplementary Material SI: Eligibility criteria

Inclusion criteria:

This study aims to recruit immediate and extended family members who meet the following inclusion criteria;

1. Family has a child who experienced recurring hospitalisation with a chronic medical illness.

2. Child is not currently in hospital.

3. Family and child are residents in Northern Ireland.

4. Hospitalised child must have at least one sibling between the ages of 4 and 18. (The minimum age requirement to participate in the study will be age 4 and above. This is due to the necessity for participants to be able to engage with the interview process and communicate effectively).

5. More than one caregiver involved in caring for the sibling(s).

6. Child Sibling who is able to communicate and willing to take part in the interview process.

7. English Speaking

Exclusion criterion for the study includes:

1. Families who currently have a child in hospital,

2. Families or persons with a significant mental health history or currently receiving mental health support.

3. Families experiencing end of life care,

4. Families or persons who do not wish to participate. Families and individuals who are deemed unsuitable to participate in the study will be provided with a list of resources to contact for support.

5. Families who reside outside of Northern Ireland.

Rationale for age threshold (4-18 years);

It has been documented within child development literature through meta-analyses, that children age 4 and above can recognise the emotions of others, both in person and through the use of photographs and other visual supports (Shaffer et al., 2019). Therefore, the minimum age requirement to participate in this study is 4 years old. This study is inclusive of all child siblings up until the age of 18. Thereafter, any siblings do not classify as a child sibling and therefore do not meet criteria to participate in the study. Children aged 16 years and above will be provided with adult consent forms to complete and be provided with a choice of having an adult present or completing the interview independently.

Supplementary Material SJ: QUB Policies

QUB Health and Safety

The researcher, Lauren Murray, has completed health and safety training as part of her role as a trainee clinical psychologist. Health and Safety policies will be closely adhered to throughout all aspects of the research and can be found via the School of Psychology Staff Hub - Health, Safety and Wellbeing Handbook.pdf - All Documents (sharepoint.com).

QUB Safeguarding

The researcher, Lauren Murray, has completed Health and Social Care training on recognising and responding to safeguarding concerns for children and young people. The research team has a duty to respond promptly and effectively if they suspect that child/ children are in need of safeguarding support services, as defined by the Children (NI) order.

If safeguarding concerns are identified throughout this research, the researcher, Lauren Murray will;

1. Discuss concerns with the research team, containing qualified Clinical Psychologists and follow advice.
2. Records are maintained in relation to all practice, liaison, decisions and outcomes in line with data protection principles and records management legislation.
3. QUB Safeguarding Policy will be followed throughout the study and can be accessed through https://qubstudentcloud.sharepoint.com/sites/int-peopleandculture/SitePages/Safeg.aspx

QUB Lone Working

QUB lone working policy will be adhered to (See below). A 'Buddy System' will be used for lone working purposes. This will involve Lauren Murray (LM), contacting the research team via telephone prior to and post interview to ensure safety for both the researcher and participants. This will also allow for any issues or concerns to be discussed.

Version: 1

Dated: November 2010

QUEEN'S UNIVERSITY BELFAST

Health and Safety Policy and Guidance Note

Lone Working/Working in Isolation

1 Lone Working Policy and Guidance

Staff and students may be permitted to work alone or in isolation provided appropriate precautions have been taken and the risks are not unacceptable. Hazardous work activities associated with significant risks should not be undertaken in isolation except where there are appropriate measures in place to mitigate the risk.

1.1 Categories of hazardous work activity with significant risks may include work with:

- dangerous machinery or equipment;
- high-voltage electrical systems;
- dangerous chemicals;
- pressure systems;
- large or potentially dangerous animals;
- dangerous pathogens;
- sources of ionising and non-ionising radiations;
- cryogenic materials;
- hot substances or equipment;
- cutting tools or implements or other sharp objects.

1.2 Certain categories of hazardous work shall <u>not</u> be undertaken by staff or students in isolation under any circumstances. These include:

(i) any work involving entry into a confined space or other area where there is a reasonably foreseeable risk of exposure to asphyxiating or toxic gases or vapours or conditions resulting in oxygen deficiency;

(ii) any work on fragile roofs or at high levels where measures to guard against falls are not provided;

(iii) any work entailing entry into or access onto any building or structure in the course of demolition or erection or any workings above or below ground level where there is a reasonably foreseeable risk of collapse or other failure.

1.3 Before any work in isolation is undertaken the risks to health and safety must be assessed by management and, where significant risks exist, they must be documented. Before work in isolation which has significant risk commences, permission must be given by senior management and a safe system of work agreed and implemented.

1.4 Safe working arrangements must also include the identification of the action to be taken in the event of an accident or emergency. Any person working in isolation that becomes aware of or encounters an unforeseen hazard should stop work (as long as it is safe to do so), and leave the area if necessary. Advice or assistance should be sought from their Supervisor or Security, if their Supervisor is not available.

1.5 The medical fitness of staff and students should be taken into account before permission to work in isolation is granted. Medical conditions which are likely to require urgent medical attention, such as some forms of diabetes or epilepsy, may preclude working in isolation. Schools and Directorates should contact the University's Occupational Health Service for advice, where necessary. In all cases emergency arrangements should be put in place.

1.6 During normal working hours it may be considered necessary for lone workers undertaking work involving significant risk to contact the University Security Control Room (Tel: 5099) to have their presence recorded as a point of first contact in the event of any security or safety issue. This requirement will be identified in the risk assessment. Out of hours Security must be informed (see below). Where appropriate, further calls can be agreed with the Control Room staff to periodically check on welfare. When a Security Patrol is in the area they may check in with the member of staff to ensure they are safe.

QUB Field Working

Due to the possibility of the research interviews taking place in CHC premises, the QUB field working policy will be closely adhered to.

FIELDWORK GUIDANCE

UNIVERSITY SAFETY SERVICE

Policy Number	
Version Number	1
Approval Date	07/03/14

Approved By	
Review Date	
Lead Responsibility	Safety Service
Lead Author	J McConkey

1. GENERAL

Queen's University Belfast is committed to protecting staff and students participating in fieldwork activities by assessing, controlling and reducing the potential risks, so far as is reasonably practicable.

2. Scope

The Health and Safety at Work Order for Northern Ireland requires employers to protect the safety and health of employees and others associated with its activities. The Health and Safety Management Regulations require employers to carry out risk assessments of activities, to document those with significant risk and put in place suitable and sufficient controls to eliminate or reduce the risks.

USHA/UCEA Guidance on Safety in Fieldwork was drawn up in 2011 to provide guidance to the University sector on management of fieldwork activities. The guidance aligns good practice in the HE sector with British Standard BS 8848.

The definition of fieldwork within the guidance is:

'Any work carried out by staff or students for the purposes of teaching, research or other activities while representing the institution off-site'.

3. Outline requirements

The overall aim is to protect staff and students from injury, and to meet the requirements of the legislation and UCEA guidance, by assessing the risks associated with fieldwork activities and reducing the risk of injury to the lowest reasonably practicable level. This will be achieved by ensuring:-

- management procedures for authorising fieldwork are in place

- risk assessments of fieldwork activities are carried out

- safe systems of work are established

4. Responsibilities

Heads of School/Directors have a responsibility to ensure that arrangements are in place to manage fieldwork activities in areas under their control.

Placement organisers, fieldwork leaders and independent field workers have responsibility to ensure that planning is done at a local level, that risks are considered and controlled to minimise the risk of injury, so far as is reasonably practicable, and that safe systems of work are adhered to.

Staff and students must adhere to any identified safe working practices put in place to manage risks from fieldwork activities. Any failures in systems or practices should be reported promptly.

Staff and students must inform their line manager/fieldwork organiser if there is any health problem or condition that might affect them during fieldwork, and comply with any health advice given by the University's Occupational Health Service.

Staff with responsibility for fieldwork must ensure that staff and students are made aware of this guidance, that they understand it and are able to comply with it.

5. Assessment Procedure

5.1 Fieldwork Risk Assessment

All activities associated with fieldwork off-site should be considered as part of a general risk assessment, and a determination of low, medium or high risk should be made. Sample risk assessments and a risk assessment format are provided (Supplementary Material 1).

5.2 Low risk activities

Further detailed assessment is not required where fieldwork activities are identified as low risk. A generic risk assessment will be sufficient. Arrangements for routine travel should comply with the Travel Safety Policy and Guidance.

Examples of low risk fieldwork activities off-site:

Attendance at conferences, visiting educational institutions, participating in recruitment fairs, etc. in the UK/EU/USA.

5.3 Detailed assessment

Where the general assessment identifies that field work activities present a medium or high risk then a more detailed assessment needs to be carried out by a competent person. Consideration should be given to measures required before, during and after fieldwork. Practical risk reduction measures should be implemented to reduce risks to the lowest reasonably practicable level.

Examples of medium risk fieldwork activities off-site:

Conducting social science interviews, organised routine student field trips, field workers / students with significant medical conditions, etc.

Examples of high risk fieldwork activities off-site:

Lone working in remote locations, work in extreme climates, work in areas of endemic and epidemic disease, activities which pose unusual hazards, travel to areas of political instability, etc.

5.4 Review

Fieldwork risk assessments should be regularly reviewed and amended where appropriate, and records of risk assessments and training should be maintained.

5.5 Accidents and Injuries

All accidents and injuries related to fieldwork activities must be reported using the University’s Accident Reporting procedure, be suitably investigated, and risk assessments reviewed.

6. Information, Instruction and Training

Staff and students must be provided with adequate information, instruction and training to enable them to carry out fieldwork activities safely. This will include awareness of risk assessments, safe working procedures and emergency arrangements.

Staff must be suitably competent and assessments should identify in broad terms the foreseeable problems likely to arise and the measures needed to deal with them. Staff training on risk assessment and management systems required for fieldwork is provided by the Occupational Health and Safety Service.

7. Record Keeping

Records of risk assessments and the provision of any instruction and training must be kept. Records should be retained for at least three years.

8. Monitoring/Audit

Schools and Directorates should monitor compliance with this guidance and take appropriate action where necessary. Records of risk assessments, safe working procedures, instruction and training etc. should be made available for audit.

9. Regulations & Guidance

The Management of Health and Safety at Work Regulations (NI) 2000

USHA/UCEA Guidance on Health and Safety in Fieldwork 2011

BS 8848: Specification for the provision of visits, fieldwork, expeditions and adventurous activities outside the UK BSI 2009

Supplementary Material SK: Consent forms

QUEEN'S UNIVERSITY BELFAST

NI Children's Health Coalition

|

Declaration of Informed Consent

Name: ____. Initials

1. I agree to participate in this research. ....

2. This agreement is of my own free will. ....

3. I have been given information about this research and aware of what my participation involves and I have received the researcher's name and contact details if I require further information. ....

4. I have had the opportunity to ask any questions about the study. ....

5. I realise that I may withdraw from the study immediately and within a 4 week time period, without having to give a reason and without consequence ....

6. I am aware that even after participating, I can decide to withdraw my data up until the time specified on the participant information sheet (4 weeks after interview date). ....

7. I understand that all personal information provided by myself will remain confidential and no information that identifies me will be made publicly available. ....

8. I am aware that any information I disclose that suggests any harm or risks to safety, the researcher is required to share this information to get me and my family the best support available. ....

Signed: .... Date:.................................

(by participant)

Print name: ....

Signed:... Date:..............................

(on behalf of researchers)

QUEEN'S UNIVERSITY BELFAST

Child Assent Form

Hi my name is Lauren

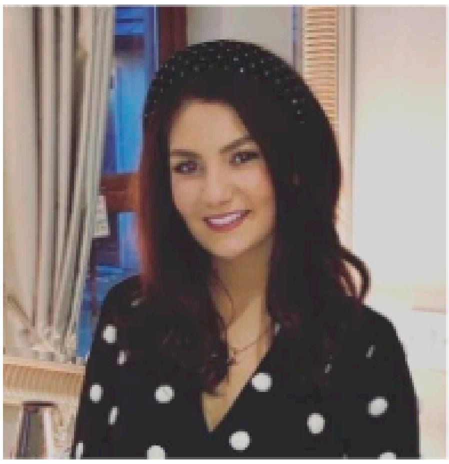

I would love to spend some time with you and talk to you about (name of child) being in hospital. I would really like to know what you thought about all of this.
If you are happy to talk to me, your (Name of person e.g Mum, Dad, Granny etc) will be able to stay with you in the room and you can tell me or (name of person) if you would like to stop and leave the room.
I won't keep you any longer than 30 minutes!

If you are HAPPY to have a chat with me, please circle the Green ✓

If you do NOT want to have a chat with me, please circle the RED X

✓	✗
YES, I WILL CHAT TO LAUREN	NO I DON'T WANT TO CHAT TO LAUREN

Date:__________
Signed by child: ______________________________
Signed by Parent: ______________________________
Signed by Researcher: ______________________________

Research Poster

Exploring the impact of child hospitalisation on the family system

A qualitative study using framework analysis

QUEEN'S UNIVERSITY BELFAST

NI Children's Health Coalition

Authors

Lauren Murray, Trainee Clinical Psychologist, Queens University Belfast

Professor Pauline Adair, Queen's University Belfast

Professor Nicola Doherty, Department of Health (DoH)

Affiliations

NI Children's Health Coalition

Introduction

Existing research on child hospitalisation has primarily focused on the perspectives of children with Chronic Health Conditions (CHCs) and their primary caregivers during periods of hospitalisation (Ravindran & Rempel, 2010). This early research illuminated gaps within familial experiences of child hospitalisation, reinforcing the importance of broadening the scope of exploration.

Research emphasies that child hospitalisation can have profound implications of emotional distress, practical, financial and social disruptions for all family members. Exploring the experiences and realities of child hospitalisation for main caregivers, extended caregivers and child siblings aims to understand the impact and challenges involved to inform healthcare practices and design interventions to mitigate the impacts of hospitalisation for affected families.

Research Questions

What are the experiences of main caregivers, extended caregivers and child siblings facing child hospitalisation?

How are these experiences similar and/or different?

What are the positive and negative impacts on the family system?

What is the specific impact on a child siblings' daily life, communication and relationships with family members and others?

Methodology

Semi-structured interviews were designed and conducted. Eight families participated in the research. Participants comprised of primary caregivers (n=8), all of whom identified as mothers, extended caregivers (n=7), including fathers and grandparents, and child siblings (n=8), aged between 4 and 16 years. Participants all resided in Northern Ireland and met eligibility criteria for the study.

Themes identified

- Emotional Impact
- Practicaities
- Relational Impact
- Take home messages
- Adjustment
- Communication

Analysis

- The interviews underwent analysis employing the Framework Method (Gayle et al., 2013, Ritchie & Spencer, 1994).
- Using the Framework Method facilitated a systematic exploration of the experiences shared during the interviews, uncovering patterns and insights relevant to the impact of child hospitalisation on the family systems from varying familial perspectives.
- Each family completed separate interviews, totalling three interviews per family and amounting to 23 datasets collected.
- Interviews were recorded and transcribed using Microsoft Teams application.
- The Framework Method steps were employed and followed.
- Six overarching themes were identified (Emotional impact, Relarional Impact, Practicalities, Adjustment, Communication and 'Take-home' messages from families.

Conclusion

Research findings aim to inform services and policy by uncovering the impacts of child hospitalisation on family systems. By identifying implications, gaps in support, access disparities, and communication barriers, targeted clinical interventions and preventions can be implemented to empower families and healthcare professionals. Ultimately, this research promotes a deeper understanding of the challenges of child hospitalisation, fostering empathy and highlighting the need for improved supports in paediatric healthcare internationally.

Call to Action

Theoretical frameworks

Attachment Theory (Bowlby 1969)

Systems theory (Checkland, 1999)

Bronfenbrenner's ecological model (1979)

Theory of Cognitive Development, (Piaget & Cook, 1952)

References

Bowlby, J. (1969). Attachment and loss (No. 79). Random House.

Bronfenbrenner, U. (1979). The ecology of human development: Experiments by nature and design. Harvard university press.

Checkland, P. (1999). Systems thinking, systems practice.

Gale, N. K., Heath, G., Cameron, E., Rashid, S., & Redwood, S. (2013). Using the framework method for the analysis of qualitative data in multi-disciplinary health research. BMC Medical Research Methodology, 13(1), 1-8.

Piaget, J., & Cook, M. (1952). The origins of intelligence in children (Vol. 8, No. 5, pp. 18-1952). New York: International Universities Press.

Ravindran, V. P., & Rempel, G. R. (2011). Grandparents and siblings of children with congenital heart disease. Journal of Advanced Nursing, 67(1), 169-175.

Ritchie, L Spencer. (1994). Qualitative data analysis for applied policy research, p. 173-194.
